# Landscape Development During a Glacial Cycle: Modeling Ecosystems from the Past into the Future

**DOI:** 10.1007/s13280-013-0407-5

**Published:** 2013-04-26

**Authors:** Tobias Lindborg, Lars Brydsten, Gustav Sohlenius, Mårten Strömgren, Eva Andersson, Anders Löfgren

**Affiliations:** 1 Swedish Nuclear Fuel and Waste Management Co, Box 250, 101 24 Stockholm, Sweden; 2 Umeå Marine Sciences Centre, Norrbyn, Hörnefors, 910 20 Umeå, Sweden; 3 Department of Ecology and Environmental Science, Umeå University, 901 87 Umeå, Sweden; 4 Geological Survey of Sweden, Box 670, 751 28 Uppsala, Sweden; 5 EcoAnalytica, Haeffnersvägen 23, 129 36 Hägersten, Sweden

**Keywords:** Sediment dynamics, Shoreline displacement, Succession, Development, Digital elevation model, Forsmark

## Abstract

Understanding how long-term abiotic and biotic processes are linked at a landscape level is of major interest for analyzing future impact on humans and the environment from present-day societal planning. This article uses results derived from multidisciplinary work at a coastal site in Sweden, with the aim of describing future landscape development. First, based on current and historical data, we identified climate change, shoreline displacement, and accumulation/erosion processes as the main drivers of landscape development. Second, site-specific information was combined with data from the Scandinavian region to build models that describe how the identified processes may affect the site development through time. Finally, the process models were combined to describe a whole interglacial period. With this article, we show how the landscape and ecosystem boundaries are affected by changing permafrost conditions, peat formation, sedimentation, human land use, and shoreline displacement.

**Electronic supplementary material:**

The online version of this article (doi:10.1007/s13280-013-0407-5) contains supplementary material, which is available to authorized users.

## Introduction

During the Quaternary period (−2.5 million years to present), the earth has experienced up to 40 glacial cycles (Lisiecki and Raymo 2005). In the northern hemisphere, these cycles have been characterized by repeated advance and withdrawal of different biomes influenced by a changing climate. Depending on location, the impact on the landscape has been different. The process understanding of how glacial cycles may structure and distribute biota has been well described (e.g., Koca et al. 2006; Bos et al. 2009). However, the understanding of glacial cycles has not been used to describe the long-term (up to 100 000 years) effects on ecosystem distribution, especially not during a whole glacial cycle and for a specific site. There are also few studies describing the combined effects of major driving forces such as sedimentation and shoreline displacement on landscape development, particularly into the future (but see Ikonen et al. 2008; Saarse et al. 2010). The reason for this is twofold. First, it is commonly reasoned that the future is much too hard to predict and that the required assumptions are difficult to support due to process uncertainties. Second, there has been no societal need for a detailed forecast of the far future at a local scale. The need to describe possible future ranges in ecosystem distribution at specific sites has increased during the last decades, and the nuclear power industry is one of the main drivers in this research field. This is due to the commitment that nuclear energy puts on the present generation to protect future humans and the environment from harmful doses of radiation, potentially due to releases of radionuclides from repositories for spent fuel and other types of radioactive waste.

When nuclear energy emerged as a serious producer of electricity after World War II, almost no concerns were raised about the management of the spent fuel. In 1957, the first method describing long-term storage (deposition in geological salt formations) was suggested by the National Academy of Science in the US (Rechard 1999). Since then a number of possible solutions for the storage and disposal of radioactive wastes have been developed (Macfarlane and Ewing 2006). One common issue for the different solutions is the need to isolate the spent fuel from humans and environment in a repository for periods of up to several hundreds of thousands of years. To test repository methodologies and to make realistic and cautious calculations for risk assessments, the need to describe a relevant and realistic future biosphere has emerged. Methodologies have been developed to describe future biospheres (Pinedo et al. 2003; Linsley and Torres 2004; Brennwald and van Dorp 2009), but these concepts have not relied on scientific understanding of the historical development for a specific site, or physical constraints on ecosystem functions for such a site, but rather stylized globally generic biospheres. When arguing for future biospheres at specific sites to be applied in risk assessments, it is necessary to support the model simplifications and the assumptions made on data ranges for site-specific properties in time and space. To have that support, long-term process understanding is needed.

During earlier study in Sweden, we showed the possibility to give a rather detailed description of site characteristics and the historical development of a site (Bradshaw et al. 2006; Lindborg et al. 2006; Lindborg 2008). Having that material at hand, our understanding that the successional patterns at a landscape scale would, in the long run, repeat themselves was strengthened. Therefore, our hypothesis is that the major driving forces on long-term landscape development are few and can be quantitatively characterized. Moreover, we consider that these drivers can be identified by the understanding of the historical development of a specific site. For a coastal site in Sweden these drivers are climate variation, shoreline displacement, and mass fluxes of matter via water or primary production (e.g., lake infilling or peat formation). By applying the above processes on the landscape geometry (topography and bathymetry), we can model the development of ecosystems at the landscape level and the potential for future land emergence or submergence. The rationale behind the hypothesis relies mainly on previous studies (e.g., Påsse 2001; Söderbäck 2008; Brandefelt and Otto-Bliesner 2009; Brydsten 2009; Brydsten and Strömgren 2010; Kjellström et al. 2010; Brandefelt et al. 2011).

To examine the hypothesis, we specifically asked: (i) Is it possible to use the major drivers identified for global glacial cycles on a specific local site to describe the main ecosystems during landscape development? (ii) Is it possible to mimic the historical landscape development in a model and extrapolate it to the future? and (iii) Can we build a model that dynamically describes the ecosystem and landscape information needed to calculate radiation dose (from a hypothetical radionuclide release in a far future biosphere) and that develops spatially in time at a landscape level?

In steps, we describe the different features and processes involved in long-term environmental change and show how the processes change the landscape features during a whole glacial cycle. This is further discussed in terms of land use potential in future landscapes with different climates. Finally, we present a landscape development model for the Forsmark area in Sweden to be used as a base in assessments of calculating risks arising from a nuclear waste repository in relevant variants of future possible landscapes.

## Materials and Methods

### The Site

**Fig. 1:**
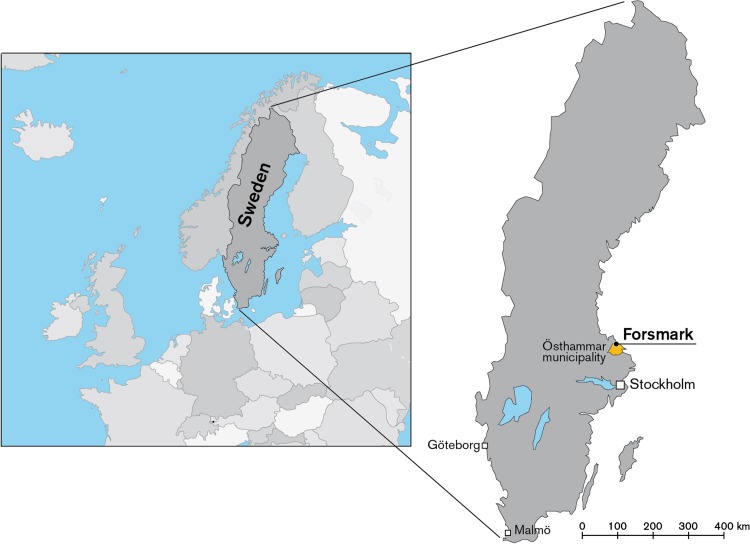
Location of the Forsmark site in Sweden. The area comprises forests, wetlands, agricultural areas, lakes, and the Baltic Sea

The Forsmark study site is located 150 km north of Stockholm at the shoreline of the Baltic Sea in the county of Uppland, Sweden (Fig. 1). Post-glacial land uplift, in combination with the flat topography, implies fast shoreline displacement that has resulted in young terrestrial and limnic ecosystems.

**Fig. 2:**
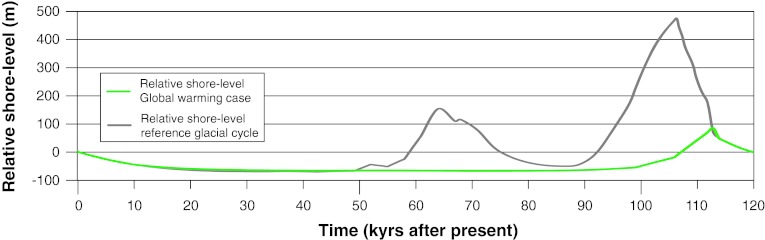
The shoreline displacement curve for Forsmark, after SKB (2010)

The latest deglaciation in Forsmark took place during the Preboreal climatic stage, c. 10 800 years ago (Strömberg 1989; Persson 1992; Fredén 2002). Forsmark is situated below the highest coastline (highest sea level after latest glaciation) and, when the latest deglaciation took place, the area was covered by c. 150 m of water at the present shoreline. The closest shore/land area at that time was situated c. 80 km to the west of Forsmark. Shoreline displacement has strongly affected landscape development, and still causes a continuous and relatively predictable change in the abiotic and biotic environments, for example in water and nutrient availability. The first parts of Forsmark emerged from the sea around 500 bc. Thus, the post-glacial development of the area has been, and continues to be, determined mainly by the development of the Baltic basin and by shoreline displacement (Fig. 2).

The study area is characterized by a small-scale topography and elevations barely exceed 20 m above sea level (ranging from −58 to +51 m a. s. l.). The model area is delimited using a future catchment area containing present land and sea. Till is the dominant Quaternary deposit (QD), whereas granite is the dominant rock type. The annual precipitation and runoff are 560 and 150 mm, respectively. The lakes are small, <1 km^2^ and shallow with maximum depths of about 2 m. Seawater still flows into the most recently formed lakes during high sea level. No major water courses flow through the central part of the site. The small brooks carry water most of the year, but can be dry for long periods during dry years. Groundwater levels in the QDs are very shallow, on average less than 0.7 m below ground for 50 % of the time (Johansson 2008).

**Fig. 3:**
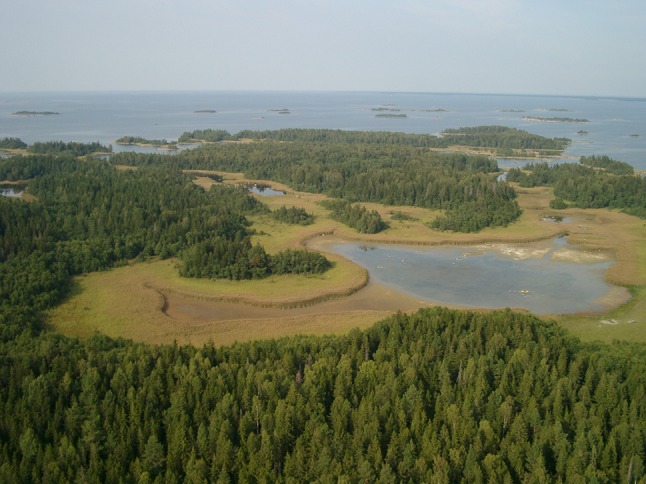
Aerial photograph of the Forsmark landscape showing the ongoing landscape succession from sea to land via young new-born lakes and wetlands

Forsmark is situated in a relatively productive coastal area in a region of otherwise fairly low marine primary production. The seawater has nutrient concentrations ranging from 330 to 790 μg L^−1^ tot-N and 12 to 25 μg L^−1^ tot-P (Aquilonius 2010). The seabed is dominated by erosion and transport bottoms with heterogeneous and mobile sediments consisting mainly of sand and gravel with varying fractions of glacial clay. The lakes in Forsmark are mainly shaped by the small topographic gradients in combination with the on-going shore displacement and short distance to the sea (Fig. 3), and by the occurrence of calcium-rich deposits. The bedrock, the properties of the QDs, and human land use affect the terrestrial vegetation. The QDs are mainly wave-washed till, but in depressions, a deeper soil layer is found, with fairly high lime content. The calcareous influence is typical for the north-eastern part of Uppland County and is among other things manifested in the abundance of rich fens. Wetlands occur frequently and cover 10–20 % of the area in the three major catchments and up to 25–35 % in some sub-catchments (Johansson 2008). A major part of the wetlands is a mix of coniferous forest swamps and open mires. The woodland is characterized by coniferous trees and has a long history of forestry. Arable land, pastures, and clear-cuts dominate the open land. The pastures were once intensively used, but are today part of the abandoned farmland following the nation-wide general regression of agricultural activities (Eriksson et al. 2002; Löfgren 2010).

### Modeling Landscape Succession

To develop a model that describes long-term landscape development, we needed to document and quantify the geometry and properties of the present ecosystems in the landscape, as well as the processes over time (Table 1). As a starting point, we used a climate model that describes the latest glacial cycle, the Weichselian, by Kjellström et al. (2010). This model gave us input to build the future shoreline displacement curve (Lindborg 2010) and to define temperature-dependent site specific climate domains needed to determine when in time Forsmark is above or below sea level during a whole glacial cycle. Using this model, we were able to confirm that the latest glacial cycle is a useful representation of glacial cycles to come (Kjellström et al. 2009; Näslund et al. 2013).

**Table 1:** The sub-models used in the landscape development model. Model extent together with model resolution can be seen. A quality classification in three levels (excellent, good, and acceptable) is shown that relies on quantitative information in the listed references or qualitative evaluations made as part of the study presented in this article

Model	Extent/resolution	Model confidence	References
Climate model	Global/Forsmark	Good	Kjellström et al. (2010)
Digital elevation model	Forsmark/20 m	Excellent	Strömgren and Brydsten (2008)
Digital elevation model	Fennoscandia/500 m	Acceptable	Brydsten (2009)
Regolith depth model	Forsmark/20 m	Good	Hedenström et al. (2008)
Regolith-lake development model	Forsmark/20 m	Acceptable	Brydsten and Strömgren (2010)
Sub-wave model	Baltic sea	Acceptable	Brydsten and Strömgren (2010)
Sub-lake model	Forsmark	Good	Brydsten and Strömgren (2010)
Sub-marine model	Forsmark	Acceptable	Brydsten and Strömgren (2010)
Shoreline displacement model	Forsmark	Good	Kjellström et al. (2010)
Shoreline displacement model	Fennoscandia	Acceptable	Brydsten (2009)

A digital elevation model (DEM) was put together from various datasets described in Strömgren and Brydsten (2008). This DEM was the basic geometry used to build a regolith depth model (RDM) (Hedenström et al. 2008). The RDM gives the depth of soils and sediments within the Forsmark area and the stratigraphic soil and sediment layers they are organized into. In this study, the term “regolith” is defined as unconsolidated material, independent of genesis, covering the bedrock. To describe biotic properties, e.g., vegetation type distribution and biomass production, we used site-specific data (Andersson 2010; Aquilonius 2010; Löfgren 2010). A coupled regolith-lake-biota development model was constructed and applied to the Forsmark area. The model consists of two main modules (see following sections): a marine module that simulates sediment dynamics (erosion, transport, and accumulation) in the sea (including the periods with freshwater in the Baltic) and a lake module that simulates lake ontogeny. Finally, the information put together using a geographical information system (GIS) was used to merge results from the two modules into a resulting set of maps (see Appendices S1 and S2 in Electronic Supplementary Material) showing the Forsmark landscape developing during the present interglacial starting from after the ice cap had disappeared (9500 bc) and into the far future (35 000 ad).

### Step by Step Approach

**Fig. 4:**
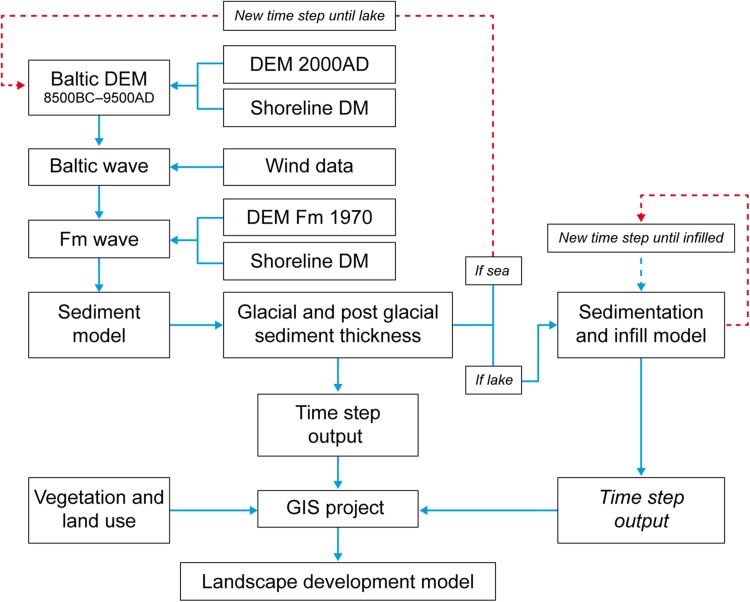
A conceptual flow chart of the coupled Regolith-Lake-Biota development model. The flow chart describes the work process and the linkage between the underlying models used for each time step to build a landscape development model at Forsmark (Fm). The marine module runs independently from the lake module and the lakes are modeled one by one due to their individual appearance in time and space. *Red dotted lines* variant indicates start of a new time step

The overall modeling strategy was to set up a chain of models that mimics the major processes involved in landscape development. In Fig. 4, the different models used, and their linkages to each other are shown. For the marine module, the whole model area and all time steps were run in one single operation. Pre- and post-processing were done in ArcGIS 10. The marine module was constructed based on input from the RDM (Hedenström et al. 2008), the shoreline displacement model (Kjellström et al. 2010), and the wave model (Brydsten 2009). A marine geology map, marine regolith depths, and a digital bathymetry model (DEM for the sea) were main outputs from the marine module for each 500-year time step. The outputs were then produced as raster layers and cover the marine part of the model area. These raster layers were later merged with outputs from the lake module and outputs from the sub-models to form continuous raster maps for the whole model area.

The task to integrate the defined processes involved a number of steps as described above. In Appendix S1 (Electronic Supplementary Material), we further describe each module and the methods that finally lead to a combined landscape development model presented in a GIS.

### Integrating Results

The marine module was processed in one single run and resulted in three raster data sets for each time step (DEM, QD, and PGC-thickness), whereas the lakes in the lake module were processed one by one. The data sets were merged in ArcGis to receive single raster data sets for each time step for the modeled lakes. In this process, the thickness of the postglacial clay (PGC) was split into a marine and a lacustrine stratum. A fourth raster data set referring to the thickness of fen peat was also produced. The result from the marine module at 10 500 ad was used for all later time steps, as the model area is above sea level after that point in time.

The marine module results were overlain with the results from the lake module and the result was a set of raster maps that dynamically showed the topography/bathymetry, soil/sediment depth, and the sea/lake/land areas in time. In a time-lapse presentation, this set of data constitutes a model describing the landscape during a typical glacial/interglacial cycle.

### Biotic Succession and Potential for Land Use

The ecosystem succession describes the effect of sedimentation and vegetation infilling in lakes and this information was distributed at the landscape level over time. The infilling of lakes is dependent on geometrical factors, initial regolith properties, the shoreline displacement, sedimentation, in-growth of vegetation and climate. In-growth by vegetation follows a general pattern for this region that is based on peat stratigraphies (e.g., Fredriksson 2004). The future distribution of vegetation was assigned in accordance with the present dominating distribution in relation to QDs, where till is dominated by needle-leaved forests, e.g., Norway spruce (*Picea abies*) and Scots pine (*Pinus sylvestris*), and thinner soils on bedrock are dominated by Scots pine. Post-glacial clays were dominated by wetlands except when humans chose to ditch areas consisting of post-glacial clay, to create agricultural land. Glacial clay, which mainly is found in the future landscape, either was populated by forests or used for agricultural purposes. In a future periglacial landscape, where the underlying QDs are assumed to be the same, the vegetation pattern was generalized from a tundra biome (e.g., Breckle 2002; Peel et al. 2007) dominated by field and ground layers. Heath land was found on more coarse-grained deposits on slopes and other more or less well-drained localities. A shrub layer was predicted to occur in the tundra environment under more wet/moist conditions, e.g., on minerogenic soils close to mires or along rivers.

The potential for a specific land use is highly dependent on the QD properties and the land use distribution was consequently dependent on the, over time, continuously changing QD development. In Forsmark, for example, the conditions for cultivation will improve drastically as the clay-rich deposits on the present sea floor are uplifted. The present land use of different QDs in the Forsmark area was first evaluated comparing land use data with QD maps. Using the modeled QD maps for Forsmark (Brydsten and Strömgren 2010) it was then possible to model the potential land use during each time step of an interglacial.

## Results

### Forsmark Landscape

**Fig. 5:**
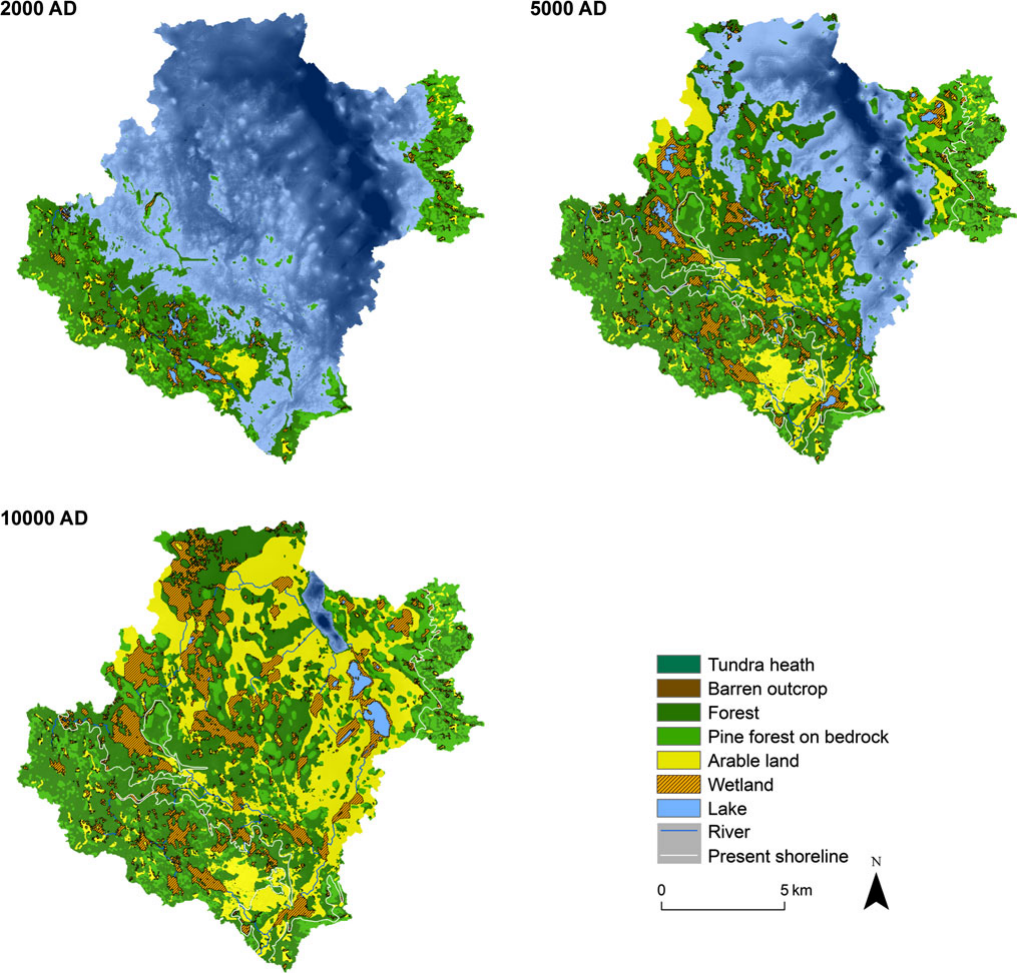
Examples from the resulting landscape development model for Forsmark at 2000, 5000, and 10 000 ad. The figure shows examples from the landscape development model variant with the land use practice that can be observed today in the Forsmark area

Climate change, shoreline displacement, and sedimentation/infilling were found to be the most important processes driving landscape development at Forsmark. The results show how the landscape develops during a glacial cycle (9500 bc to 100 000 ad) with emphasis on the interglacial and periglacial periods. The first period, just after an earlier glaciation, was characterized by sea. Land was submerged to a depth of approximately 150 m due to isostatic load from the previous ice sheet. Land emerged from the sea starting at 500 ad, and lakes and wetlands form. After 20 000 years (10 000 ad), the model area was above sea level and only a narrow bay can be seen in the northeastern part of the Forsmark landscape (Fig. 5; Appendix S2 in Electronic Supplementary Material). The vegetation and the properties needed for potential agriculture were developing in response to the developing landscape.

**Fig. 6:**
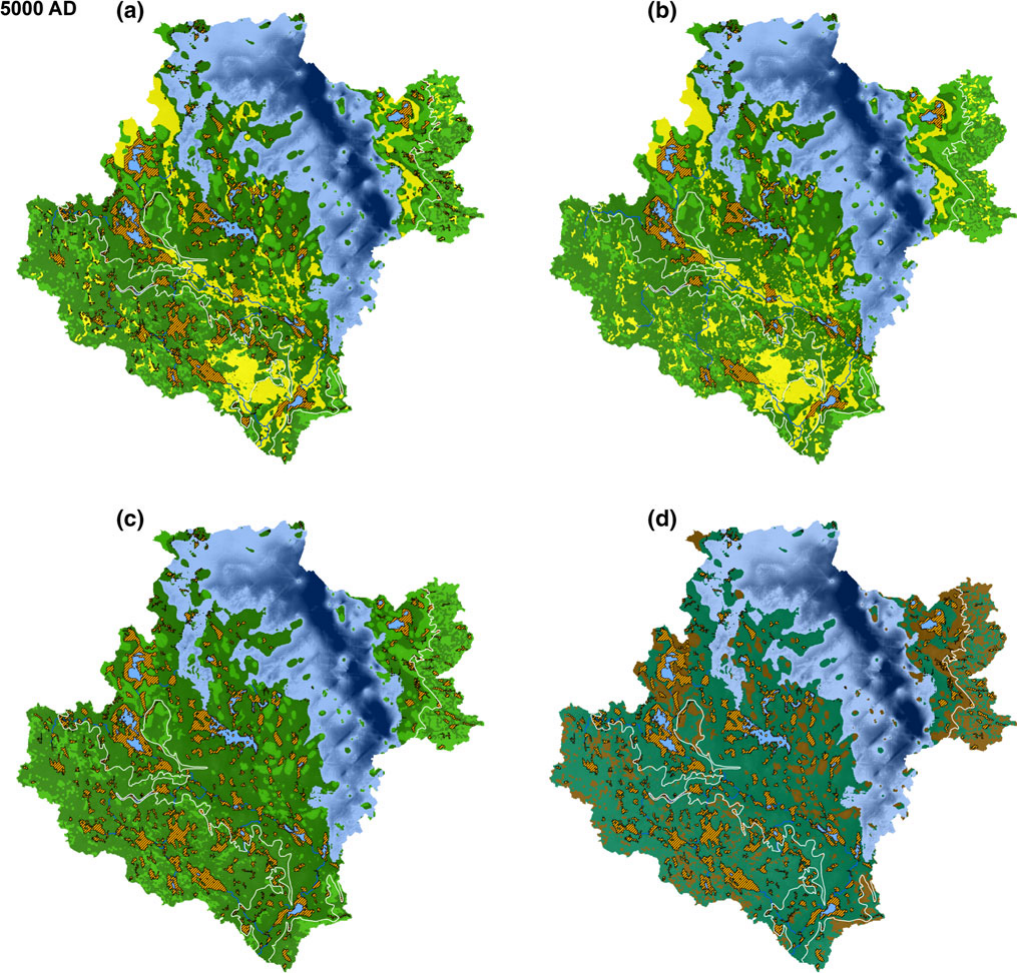
The resulting landscape development model output for a given time (5000 ad) with **a** present agricultural behavior, **b** maximum agriculture, **c** no agriculture, and **d** periglacial conditions. Color codes are the same as in Fig. 5

Variants of the landscape development can be extracted from the model. One variant illustrated the landscape development without land use, and another one showed the effects on the landscape if all possible areas were used for agriculture. In Fig. 5, a variant was illustrated that uses today’s practices in land use with present climate displayed on snapshots in time extracted from the model. Depending on climate input, the process rate in the model changes. For example, the rate of infilling of lakes slowed down during periglacial conditions, and the possibility of using areas for agriculture vanished (Fig. 6).

### Shoreline Displacement

Different kinds of landscape development were modeled in future Forsmark. When using the landscape development variant based on results from the last glacial period Weichselian climate (starting at Marine Isotope Stage 5e—the Eemian), temperate conditions will persist in Forsmark until 10 000 ad. During this period, the regressive shoreline displacement was assumed to continue, but at a gradually declining rate (Fig. 2). Initially, seabed areas will transform into new land at a rate of approximately 1 km per 1000 years. This will strongly influence the landscape, especially during the first part of the period, eventually resulting in a situation where Forsmark is located inland rather than by the coast.

The Öregrundsgrepen strait, south of the modeled area, will be cut off about 3000 ad and turn into a bay. This will negatively affect the water circulation and, due to the continuing narrowing of the bay, further restricting the water turnover. However, at the beginning of the period, turnover times are not expected to be longer than a couple of days, except for closed bays which are near isolation (Engqvist and Andrejev 2000; Eriksson and Engqvist 2013). During the period from 3000 to 5000 ad, a semi-enclosed archipelago is expected to develop northeast of Forsmark. Around 5000 ad, many straits in this archipelago will become closed and a number of lakes will become isolated from the sea. At 5000 ad, the Öregrundsgrepen bay will withdraw ca. 5 km from the present shoreline. A small stream will drain the area and some small and shallow lakes will be situated along the stream. In the period up to 10 000 ad, the Öregrundsgrepen bay shrinks gradually to finally form a short and narrow bay adjacent to the island of Gräsö.

### Sedimentation and Lake Infilling

Accumulation of sediments occurred both on bottoms at large water depths and on shallow bottoms inside the belt of the skerries which are sheltered from wave power, whereas erosion occurred mainly on shallow bottoms exposed to waves. Transport bottoms were found in all places between these two extremes, i.e., at intermediate depth with moderate wave exposure (Brydsten 2009). The amount of resuspended particles varied greatly over time and, therefore, also the sedimentation rate. In a coastal area, the main sources of resuspended particles were fluvial transport to the sea, wave-washed shores, and sea-bottom material. The fluvial input of particles is negligible compared with resuspension due to wave washing (Brydsten and Strömgren 2010) and particles resuspended due to wave washing resettling on deeper bottoms with low wave power (below the wave base). As a result of the shoreline displacement, these sediments will eventually be positioned above the wave base and resuspended again. Therefore, the wave processes were modeled to act both on postglacial fine-grained sediments and on unwashed till, using the temporal variation in sedimentation rate from Brydsten and Strömgren (2010).

The seafloor showed a characteristic evolution over time during an interglacial, beginning with a period of accumulation due to large water depth early after deglaciation. Due to decreasing water levels, the following period (2000 bc to 6000 ad) was dominated by transport and erosion. Finally, transport and accumulation in sheltered locations in the sea only occurred during a short period before the sea bottom became land, suggesting that only limited parts of the model shows continuous accumulation of sediments throughout the whole marine period. During the period from 3000 ad to 10 000 ad a large number of lakes will be isolated from the sea. Most of the new lakes were small and shallow, and will quickly transform into mires, although a few deeper lakes were projected to exist for several millennia. Around 10 000 ad, almost all lakes in the area will be infilled and only some initially relatively large and deep lakes near the island of Gräsö were expected to remain (Fig. 5).

## Discussion

### Future Climate

The climate during the temperate period may vary considerably, with both warmer and colder intervals. The main effect of temperature changes will be on the vegetation period, which today varies regionally between 170 and 210 days dependent on elevation, local topography, aspect direction, and distance to the seashore. Changed temperatures may give rise to a drier or wetter climate and to changed snow cover and frost characteristics, which in turn can affect the dominant vegetation and peat formation.

It is, however, assumed that the climate variations during the rest of the temperate period in this interglacial will not exceed the regional spatial variation and the between-year variations observed at the site today. According to the reference glacial cycle (Näslund et al. 2013), Forsmark will go through a number of climate changes resulting in temperate, periglacial (with or without permafrost), and glacial conditions. After the next glaciation, a new period of submerged conditions is also predicted. The future climate is only used as input and not modeled in this study, and below follows a short discussion on potential future land use and climate implications for ecosystems in the Forsmark landscape (further described in Näslund et al. 2013).

### Potential Future Land Use

The number of people that potentially can be sustained by food produced within the Forsmark area is strongly dependent on the distribution of different land use types (Saetre et al. 2013). The shoreline withdrawal means that the area for fishery is continuously reduced. Much of the newly formed land will be unsuitable for farming due to boulder- and stone-rich deposits, but there are significant parts with fine-grained sediments in the area today submerged under the sea. If not cultivated, most of the new land is expected to be suitable for pasture and also for forestry (Löfgren 2010). The food productivity is much higher in agricultural areas than in aquatic or non-cultivated terrestrial areas. Accordingly, the potential food productivity in the total modeled area is expected to increase due to an increasing proportion of arable land as new land areas are formed.

The availability of freshwater for human supply is expected to gradually increase with the decreasing marine influence in the area. New lakes and streams will form in the emerged land areas, but both the present and most of the future lakes will be relatively short lived due to their shallowness. It seems likely that the water from lakes and streams present in the future will be less suitable as drinking water due to their shallowness, and the main potential use of surface water is for irrigation.

### Temperate Climate Domain

After the initial temperate period (after 10 000 ad), a relatively short period of periglacial conditions will follow. The periglacial conditions will once again change back to temperate conditions that more or less will continue until 25 000 ad with a few periglacial interruptions. Another temperate period is expected around 40 000 ad that will last for about 5000 years. During far future temperate conditions, Forsmark will have characteristics that mimic the late parts of the initial temperate period. This means that there will be a landscape that comprises terrestrial ecosystems with few or no lakes and no sea. The terrestrial system will consist of forests, mires, and areas that could be used for agriculture. Higher altitude areas with outcrops of bedrock will possible be forested with pine.

### Periglacial Climate Domain

Periglacial periods at Forsmark are characterized by tundra vegetation and permafrost features. The precipitation is low, due to limited evaporation transporting water to the atmosphere. However, the low evaporation means that wet ground is prevalent even with decreased precipitation and the surplus water is unable to seep into the ground because of the permafrost. This results in extensive wetlands, with a lower peat accumulation rate than in a temperate situation. Even though there may be a snow cover of up to 50 cm during winter, higher areas are frequently blown free of snow, and here, intensive erosion occurs by the blowing ice crystals. The tundra is devoid of forests and the vegetation consists of herbs and shrubs, at more elevated dryer places lichens dominate and on wet ground mosses. The vegetation period is short.

Areas with taliks, i.e., unfrozen ground in regions of permafrost, are potentially areas for humans to settle. However, even if the taliks can be potential locations for human settlement, the low productivity in the permafrost region requires utilization of a large area to supply the resources needed by even a small community. The talik feature is also of interest when constructing conceptualizations of transport of matter via ground water from the deep bedrock to the surface system.

### Glacial Climate Domain

During glacial periods an ice sheet will cover Forsmark. For limited periods, the ice sheet will be thin over the site and elevated areas can protrude above the ice surface. There, lichens, grasses, or herbs may be present. On the ice surface, microbes, algae, and some insects can exist. At the ice margin, a productive aquatic community may exist that may sustain a fish population, which can be exploited by the animals living on the ice (e.g., birds, polar foxes, polar bears) and humans. The populations of vertebrates and humans are likely to migrate over large areas due to low food productivity or severe weather conditions. In most cases, a human population will probably comprise occasional visitors, due to the hostile environment and the variable ice-situation. It is possible that a human population could be present for longer periods close to the ice margin along the coast and live on fish.

### Submerged Conditions

In the reference glacial cycle developed for Forsmark and described in Näslund (2013), two periods of submerged conditions are identified. During these periods, Forsmark is covered by sea. The submerged conditions always followed directly after the ice sheet has withdrawn and the ice load has depressed the Forsmark bedrock. After the Weichselian glaciation, the first terrestrial areas appeared around 500 bc. The last areas in the Forsmark landscape that will become land are calculated to do so at around 11 500 ad. This means that the submerged conditions will have two phases, one first phase of ca 8000 years when the whole area is submerged, and one that continues for 12 000 years when the sea gradually withdraws and the land area accordingly expands. Submerged conditions are a landscape state when the processes and properties related to the marine or limnic system (aquatic ecosystem) totally dominate at Forsmark. These ecosystem types are not expected to change dramatically due to changes in climate, except for the effects of the long-term change in salinity. Therefore, the submerged future landscape could be treated as identical to the historical and present aquatic ecosystems at Forsmark.

### Model Validation

To validate a model that describes the future is, for obvious reasons, not possible. However, the input models and the handling of the processes therein can be validated. It is also possible to compare the model output of present landscape with site features of today. The landscape development model was, therefore, validated in two steps: (1) the input processes were validated for each input model or dataset, (2) the final model was run using initiation data from 9500 bc (just after the ice cap left Forsmark during the latest deglaciation) until the present day (2000 ad), and the model was compared with the site characteristics. The first validation step, process handling for each input model, is described in references listed in Table 1 together with a general quality judgment.

**Fig. 7:**
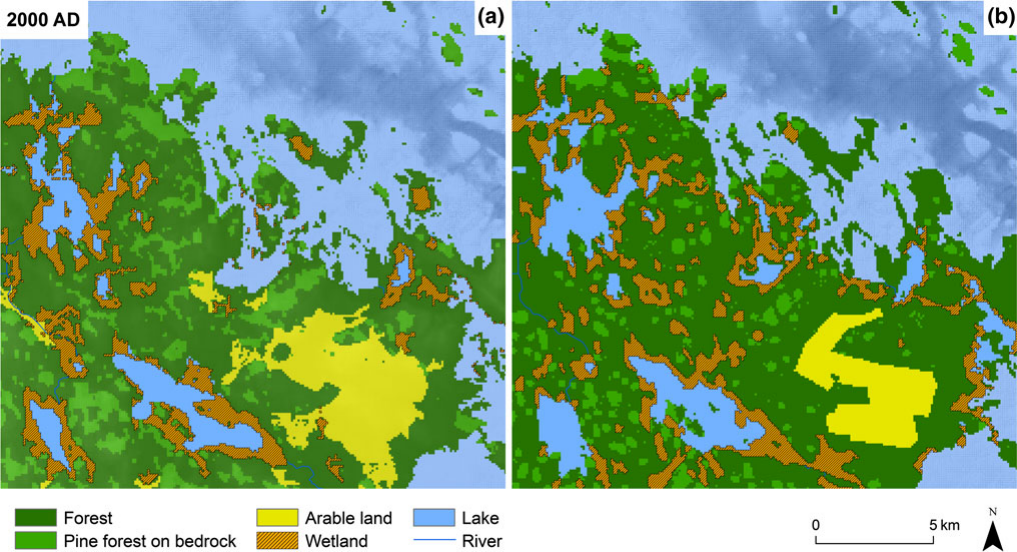
A close up of the landscape development model **a** for the year 2000 ad with present agricultural behavior compared with the present map **b** of Forsmark showing five classes of land use and vegetation

The second validation step is only qualitative and visualizes the model’s ability to mimic process rates at landscape level. In Fig. 7, the model output for 2000 ad is merged with a map of Forsmark showing present conditions at the site. It is of importance to note that the model in many ways uses the present conditions as input. The shoreline displacement rate is one example of a process that builds upon understanding of historical conditions. Therefore, the validation of the landscape development model shown here is not to be taken for a validation of the capability to model the future, but rather of its capability to mimic the historical landscape development at Forsmark. However, as long as the processes do not change dramatically, we should have a good tool for also modeling a future of relevance for the questions at issue.

In Fig. 7, the Landscape Development Model for year 2000 ad (a) is compared with the present map (b) using similar land classes as in the model. The figure shows that agricultural land use is overestimated and that wetland is underestimated in the model. One explanation for this is that the model interprets all mires with a deeper peat layer than 1 m as areas suitable for agricultural land use. Also glacial clay and fine-grained moraine are modeled as suitable for agricultural use even though today only parts of areas covered with these soils are used for cultivation. The model also slightly overestimates the infilling of lakes, but the areas covered by forest and sea is in good agreement with present conditions.

## Conclusion

The landscape development model shows that the main processes of climate change, shoreline displacement, infilling, and sedimentation are drivers that well explain the landscape development seen at Forsmark today. Although our model cannot be fully validated, we argue that the processes and features we rely upon are scientifically sound and well understood. Using the physical constraints on the Forsmark future landscape, we have narrowed down landscape variants and data from these futures and present a model that is relevant and realistic. Thus, we stress that applying the same processes to the Forsmark landscape into the future is potentially appropriate during a whole glacial cycle and beyond; especially since the confidence in the ecosystem pattern in the landscape is high. Although human activities often is regarded as having a major impact on ecosystems, we conclude that in the time frames discussed within this work, no change in long-term landscape succession is caused by human activities at the site. Nevertheless, human land use is part of the landscape development, and the type of possible land use during a glacial cycle is depending on the processes discussed in this study.

Using the present differences in elevation at Forsmark as an analog for time (due to shoreline displacement), we can argue that the current sea–lake–mire succession is a good representation of future development of areas presently submerged under the sea. This dynamic and multi-discipline approach, based on robust sedimentation and succession models, gives us a useful tool to explain the results, not only to the scientific community but also to the public as well as to practitioners searching for information on how site-specific data and understanding are extracted and used, e.g., in dose calculations. The modeling done within the framework described here is the state of the art supporting tool for predicting risks to humans and the environment, valid to use in the context of potential future releases of radionuclides from a repository for spent nuclear fuel as well as other long-term risk assessments.

## Electronic Supplementary Material

Below is the link to the electronic supplementary material.

Supplementary material 1 (PDF 16281 kb)
