# Supplementary material for: Landscape Development During a Glacial Cycle: Modeling Ecosystems from the Past into the Future
Source: Ambio. 2013 Apr 26;42(4):402–13. doi: 10.1007/s13280-013-0407-5 (PMC3636364; doi:10.1007/s13280-013-0407-5)
Supplement: Supplementary file 1 — Supplementary material 1 (PDF 16281 kb) [file 13280_2013_407_MOESM1_ESM.pdf]

Electronic Supplementary Material

Landscape Development during a Glacial Cycle: Modeling Ecosystems from the Past into the Future

Tobias Lindborg, Lars Brydsten, Gustav Sohlenius, Mårten Strömgren, Eva Andersson, Anders Löfgren

APPENDIX 1

The Marine module

The Marine Sediment Model simulates the accumulation or erosion of postglacial clay or silt particles (PGC) and generates a raster layer with thickness of PGC. The QD map and the DEM are updated based on the thickness of the PGC. This is repeated for each time step until a cell is a lake. When a lake has emerged, sedimentation and infilling processes are handled in the Lake Module (see Lake Module section below).

The Wave Model requires bathymetry (with a coarse resolution in the Baltic Sea and finer resolution in the Forsmark area) and extreme wind speed from different directions as inputs. Since a complete interglacial period is modeled (Baltic Ice Lake to future late 'Littorina' stage), the change in the Baltic Sea bathymetry over the same period must be calculated, including periods with fresh water (Baltic Ice Lake and Ancylus Lake) as well as the Ancylus transgression. Such a model for the Baltic Sea geometrical evolution did not exist, so this sub-model was also constructed within this study. Due to a gap in knowledge concerning historical and future wind conditions in the Baltic region, measured wind data for the last few decades (Brydsten 2009) were used for the whole period.

The amount of shore level displacement varies both in time and space (Påsse 2001). The Baltic Sea has also been a lake during two post-glacial periods (The Baltic Ice Lake and the Ancylus Lake), during which the eustatic process of sea-level change had no affect on the bathymetry and only isostatic processes determined the change (Påsse 2001). The Baltic Sea bathymetry model was produced in three steps. (1) A digital elevation model (DEM) for today's situation was produced. This DEM covers all land that at any time during the post-glacial period has been covered by Baltic Sea water. (2) A model that describes the difference between today's elevation and the sea-level elevation at any chosen time between 9500 BC and 7500 AD (when the shoreline displacement is taken to be complete within the model area), with the same extension and resolution as the DEM, was developed. This is called a Shore-level Displacement Model (SDM). (3) The DEM for any chosen date was calculated as the difference between the present day DEM and the SDM for the chosen date. The STWAVE program (McKee Smith et al. 2001) was used to generate waves at both regional (Baltic) and local high resolution (Forsmark) scales. The output from the high resolution wave model is sea bottom type divided in accumulation, transport and erosion in 20 meter resolution raster format. To calculate the wind speed from different directions a data set with geostrophic wind data was analyzed (Brydsten 2009).

Running the Marine Module

Having the Baltic Sea DEM, we applied it with the Wave Model for different wind forces and wind directions for the entire Baltic Sea. The data input needed was a Baltic Sea DEM for each time step (9500 BC-7500 AD) with 1 km resolution. The Wave Model produced wave height and period for each grid cell in the Baltic Sea DEM for each time step during the interglacial period. The Baltic Sea wave results were extracted for the northern part of the Forsmark model area and used as input to the local Forsmark DEM with 20 meter grid cell resolution for each time step. The Forsmark DEM was then combined with wave data from the Baltic Sea and with different wind forces and directions for the Forsmark area and for all time steps.

The most powerful wave for each pixel/time step from this simulation was saved and used as input to the sedimentation model. The sedimentation model calculates maximum orbital water velocity (U_{max}) above the sediment beneath the wave (Komar and Miller 1973). If U_{max} was below the maximum value for accumulation, a certain amount of sediment was added to the specific pixel. The amount varied for each time step as described in Brydsten and Strömgren (2010). The critical values for accumulation/erosion of postglacial fine-grained sediments (clay and silt) and erosion of glacial clays were calibrated by comparison with the present day situation at Forsmark (Hedenström and Sohlenius 2008).

Starting just after the latest deglaciation, the Sedimentation Model was run. The Soil Depth Model with all postglacial clay excluded was assumed to mimic the situation just after the deglaciation, and was the starting point for the DEM development. The postglacial clay thickness for the modeled area, pixel by pixel, was then saved and used as input to the next time step. For each time step the DEM and the thickness of the postglacial sediment was saved. During this simulation, data were saved and not changed once a pixel was found within a lake that

was isolated from the sea. These areas were instead further treated in the Lake Module.

The Lake Module

A statistical analysis of sediment cores from six lakes in the Forsmark area showed that the lacustrine sedimentation rate depends primarily on lake water volume (Brydsten 2006a). This means that the sedimentation rate decreases over time as the water volume decreases due to infilling processes. The shorelines of existing lakes were mapped with GPS and the bathymetry measured with echo soundings (Brunberg et al. 2004). The extensions and bathymetry of future lakes were modeled with the GIS 'fill tool' using the 1970AD DEM as input (Brydsten 2006a).

The GIS-based method gives a large number of future lakes, most of them very small and shallow. These small and shallow areas are not of importance for long-term landscape development since they are short lived (<500 years) and have had no role in land use historically.

The small (0.08 km^2), and today existing, Lake Puttan was, therefore, used as a reference for minimum future lake size and smaller areas in the model were omitted from further analysis. 41 lakes were identified using the above definition; 31 of these lakes will appear in the future and 10 of these lakes exist today.

Running the Lake Module

Each lake was modeled separately. Input was the Marine Module results from postglacial sediment thickness for the time step just before lake isolation for the specific lake area and the DEM for the same time step. The module calculates both sedimentation of minerogenic material (clay and/or silt) and infilling by growth of vegetation and is based on site-specific data on present lakes. Infilling with vegetation starts in the marine stage on bottoms that are shallower than 2 meters and in the future will become lake bottoms (Komulainen et al. 2008).

During the lake stage, the infilling starts with colonization by littoral plants, e.g., *Phragmites australis*, and this is followed by colonization by other peat-forming taxa such as *Carex* and *bryophytes* (Fredriksson 2004). Data from mapping of vegetation extents in 25 lakes in the Forsmark area (Brunberg et al. 2004), showed that the 'rate of infill' in lakes depends primarily on lake area. The rate of infill is a maximum value. Lower values can apply if the area of the bottom shallower than two meters is not large enough, as this is the depth that limits colonization.

The Lake Module was run until the whole lake was completely terrestrialized and no more lake sedimentation could occur. The Lake Module uses input data from the Climate Model and lowers the sedimentation and infilling rates during periglacial conditions (Brydsten and Strömgren 2010). Also Talik features (unfrozen groundwater beneath the lake) are modeled based on the work described by Hartikainen et al. (2010).

Post-processing

Since only the largest lakes are processed in the Lake Module, the DEMs hold small local depressions that are the non-processed lakes. These small lakes are assumed to be infilled during one time step (500 year) and will therefore be filled with peat. Consequently, it is necessary to process the lakes that do not show in the Lake Module. This post processing is performed for each time step using the GIS function 'fill' on the DEM from the merged data set. The fill function gives the thickness of peat at all cells in the model area, but only cells that have become land since the last time step are used. To select these cells, a land/sea raster layer was used. The layer with thickness of 'additional peat' was used to update the DEM, QD, and peat-thickness layers.

As a final step in the post-processing, wetlands not originating from infilled lakes are added. These wetlands are modeled with a Topographical Wetness Index (TWI) calculated in the GIS from the DEM (Beven and Kirkby 1979).

High TWI values mean wet conditions and are associated with a large upslope area and a low gradient. The continuous TWI raster layer is reclassified as dry/wet with a limiting TWI value of 13.2 (Brydsten 2006b). Wet areas according to TWI that have emerged from the sea during earlier time steps in the modeling are classified as hanging wetlands (primary mire formation). Peats in hanging wetlands are assumed to be thin. The thickness is therefore approximated to zero. As a consequence, the DEM is not updated for peat thickness between time steps.

APPENDIX 2

Landscape development

The landscape development in the Forsmark area is modeled in the 35 maps below for a typical glacial/interglacial cycle from 9000 BC, when the ice sheet had receded from the area and the site was completely covered by sea, to 35000 AD, when the landscape has come to a maturity stage and will be relatively stable until the next glacial stage (ca. 57000 AD).

Four different variants are modeled: 1) a land use similar as present, 2) a landscape where all areas that can be cultivated are used as arable land, 3) a landscape unaffected by humans, and 4) a permafrost variant. Variant 1, 3 and 4 is only modeled for three time steps: 2000, 5000, and 10000 AD. Variant 2 is modeled in two time steps during the sea stage and in 500 year time steps from 1500 BC to 12000 AD, and thereafter in 5000 year time steps from 15000 AD to 35000 AD. Input data for the modeling of the land-use development in Forsmark is produced in a coupled regolith- lake biota development model constructed by Brydsten and Strömgren (2010). The model consists of two main modules: a marine module that simulates sediment dynamics (erosion, transport and accumulation) in the sea (including the periods with fresh water in the Baltic) and a lake module that simulates lake ontogeny. Raster layers produced from these modules, data from a survey performed by Hedenström and Sohlenius (2008), and data produced by Hedenström et al. (2008) were used for further modeling of the land-use development in Forsmark.

Map algebra in the Spatial Analyst Tools in ArcGis 9.3 was used for all calculations.

REFERENCES

Beven, K. J., and M. J. Kirkby. 1979. A physically based, variable contributing area model of basin hydrology. *Hydrological Sciences Bulletin* 24: 43–69.

Brunberg, A. K., T. Carlsson, P. Blomqvist, L. Brydsten, and M. Strömgren. 2004. Forsmark site investigation: Identification of catchments, lake-related drainage parameters and lake habitats. Svensk Kärnbränslehantering AB, SKB P-04-25, Stockholm, Sweden, Report, 147 pp.

Brydsten, L. 2006a. A model for landscape development in terms of shoreline displacement, sediment dynamics, lake formation, and lake choke-up processes. Svensk Kärnbränslehantering AB, SKB TR-06-40, Stockholm, Sweden, Report, 33 pp.

Brydsten, L. 2006b. Modelling groundwater discharge areas using only digital elevation models as input data. Svensk Kärnbränslehantering AB, SKB TR-06-39, Stockholm, Sweden, Report, 20 pp.

Brydsten, L. 2009. Sediment dynamics in the coastal areas of Forsmark and Laxemar during an interglacial. Svensk Kärnbränslehantering AB, SKB TR-09-07, Stockholm, Sweden, Report, 65 pp.

Brydsten, L., and M. Strömgren. 2010. A coupled regolith-lake development model applied to the Forsmark site. Svensk Kärnbränslehantering AB, SKB TR-10-56, Stockholm, Sweden, Report, 52 pp.

Fredriksson, D. 2004. Forsmark site investigation: Peatland investigation Forsmark. Svensk Kärnbränslehantering AB, SKB P-04-127, Stockholm, Sweden, Report, 47 pp.

Hartikainen, J., R. Kouhia, and T. Wallroth. 2010. Permafrost simulations at Forsmark using a numerical 2D thermo-hydro-chemical model. Svensk Kärnbränslehantering AB, SKB TR-09-17, Stockholm, Sweden, Report, 142 pp.

Hedenström, A., and G. Sohlenius. 2008. Description of the regolith at Forsmark. Site descriptive modelling SDM-Site Forsmark. Svensk Kärnbränslehantering AB, SKB R-08-04, Stockholm, Sweden, Report, 221 pp.

Hedenström, A, G. Sohlenius, M. Strömgren, L. Brydsten, and H. Nyman. 2008. Depth and stratigraphy of regolith at Forsmark. Site descriptive modelling, SDM-Site Forsmark. Svensk Kärnbränslehantering AB, SKB R-08-07, Stockholm, Sweden, Report, 105 pp.

Komar, P. D., and M. C. Miller. 1973. The threshold of sediment movement under oscillatory water waves. *Journal of Sedimentary Petrology* 43: 1101-1110.

Komulainen, M., P. Simi, E. Hagelberg, I. Ikonen, and S. Lyytinen. 2008. Reed energy – Possibilities of using the Common Reed for energy generation in Southern Finland. Turku University of Applied Sciences, Report 67, Turku, Finland, 78 pp. Retrieved January 6, 2013, from http://julkaisut.turkuamk.fi/isbn9789522160355.pdf

McKee Smith, J., A. R. Sherlock, and D. T. Resio. 2001. STWAVE: Steady-State Spectral Wave Model. User's Manual for STWAVE, Version 3.0. Coastal and Hydraulics Laboratory U.S. Army Engineer Research and Development Center. Retrieved January 6, 2013, from http://chl.erdc.usace.army.mil/chl.aspx?p=s&a=PUBLICATIONS;39

Påsse, T. 2001. An empirical model of glacio-isostatic movements and shore-level displacement in Fennoscandia. Svensk Kärnbränslehantering AB, SKB R-01-41, Stockholm, Sweden, Report, 59 pp.

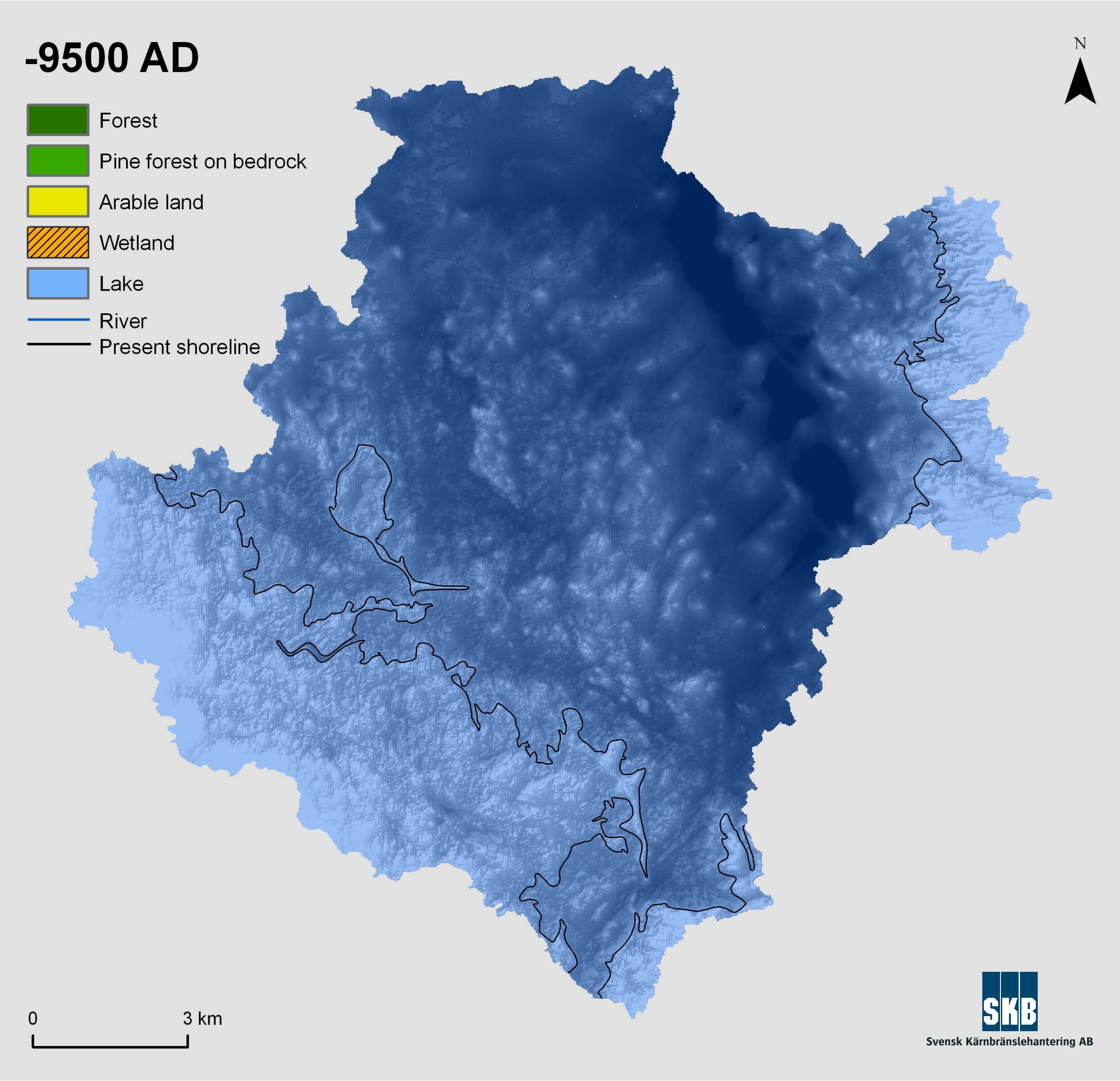
-9500 AD
Forest
Pine forest on bedrock
Arable land
Wetland
Lake
River
Present shoreline
N
0
3 km
SKB
Svensk Kärnbränslehantering AB

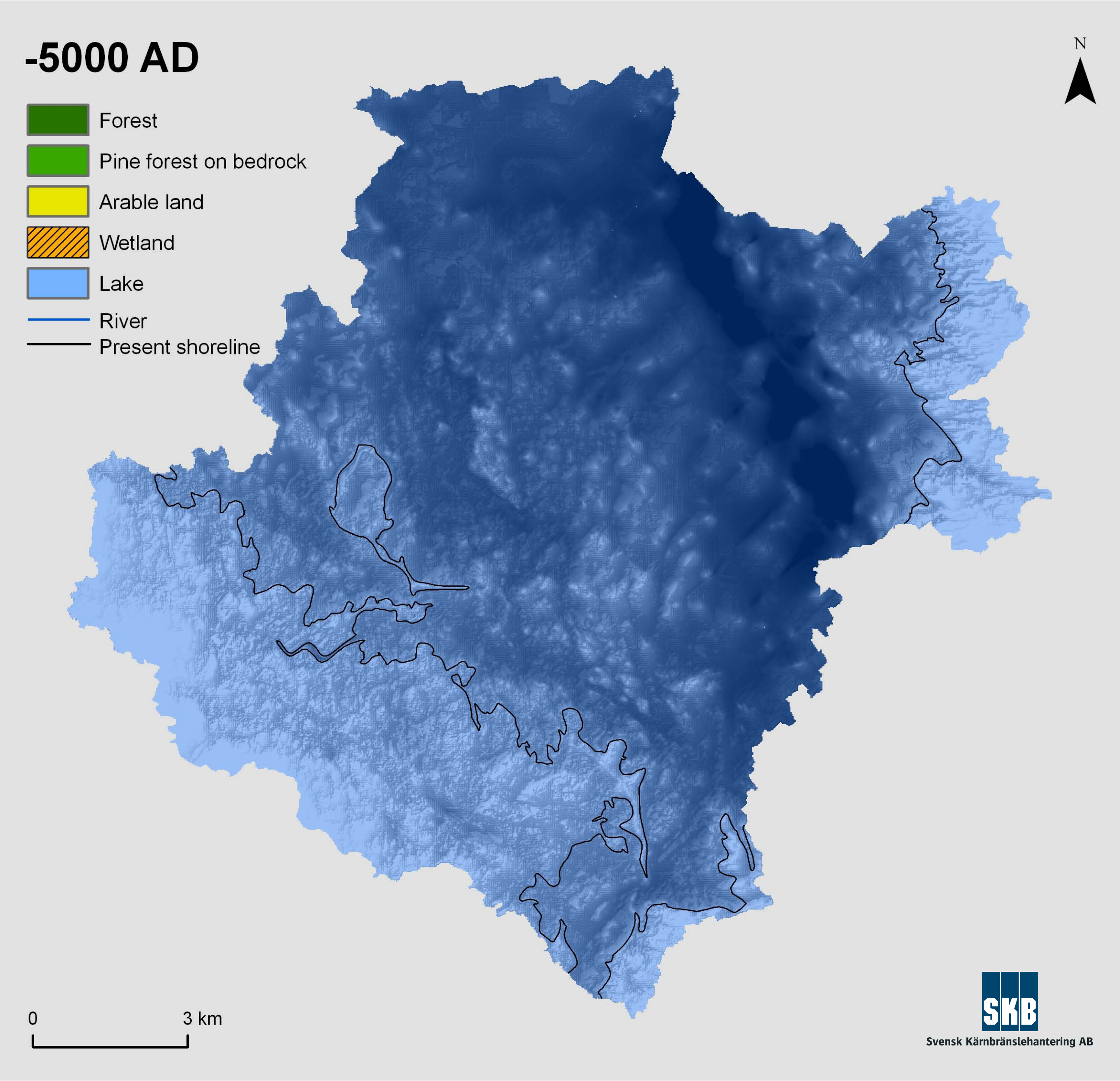
-5000 AD
Forest
Pine forest on bedrock
Arable land
Wetland
Lake
River
Present shoreline
N
0
3 km
SKB
Svensk Kärnbränslehantering AB

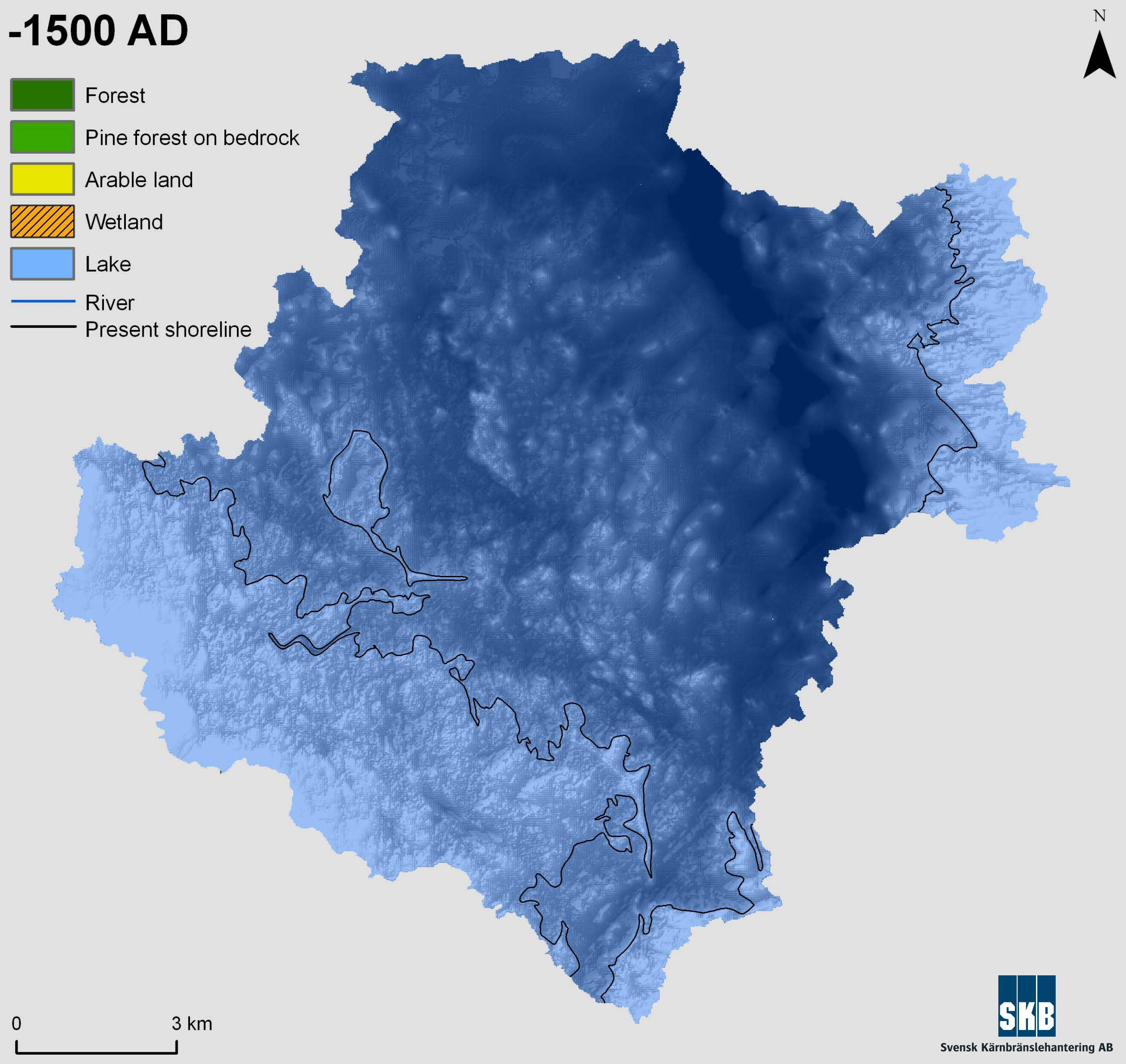

-1500 AD
Forest
Pine forest on bedrock
Arable land
Wetland
Lake
River
Present shoreline
N
0
3 km
SKB
Svensk Kärnbränslehantering AB

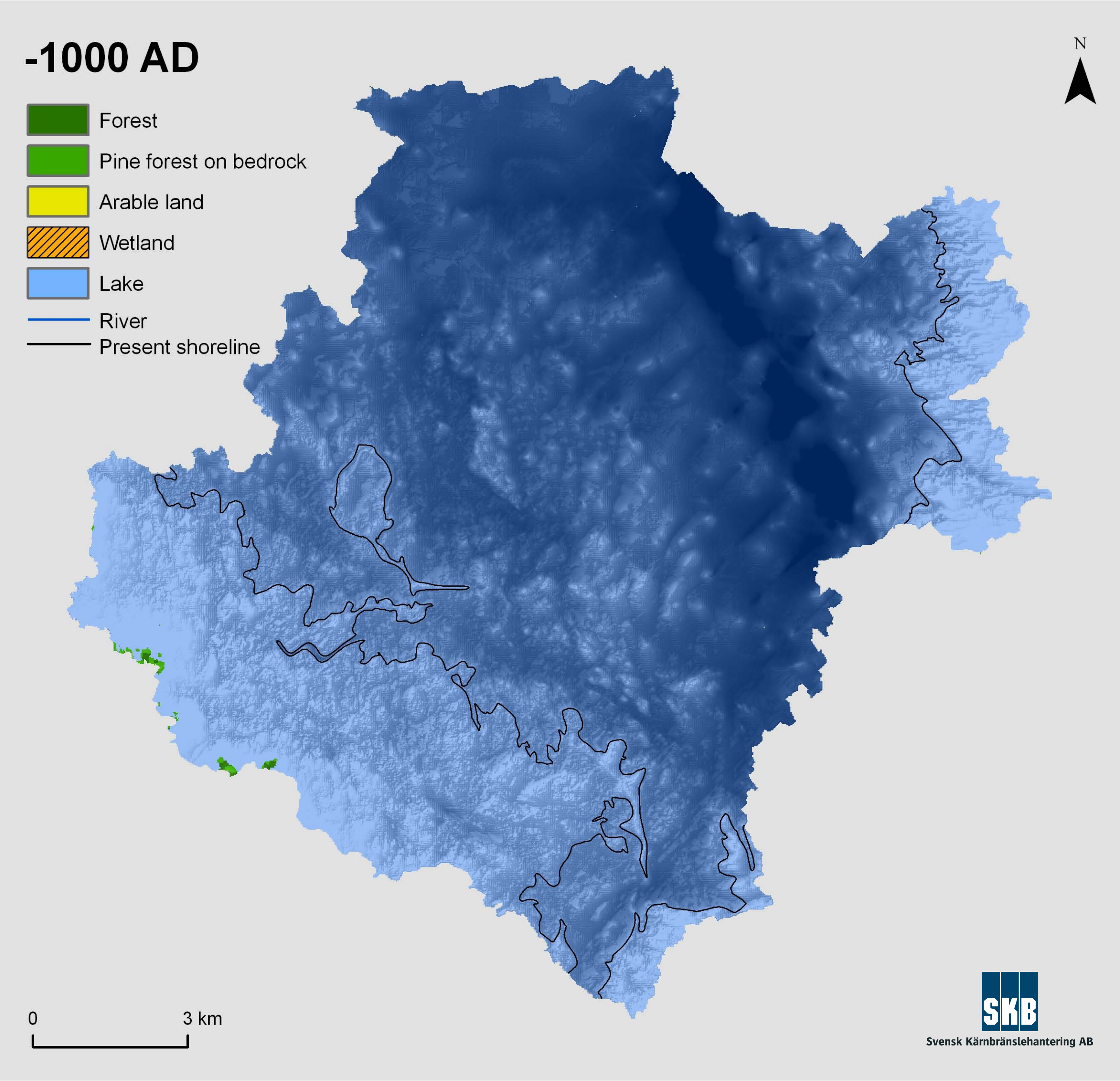
-1000 AD
Forest
Pine forest on bedrock
Arable land
Wetland
Lake
River
Present shoreline
N
0
3 km
SKB
Svensk Kärnbränslehantering AB

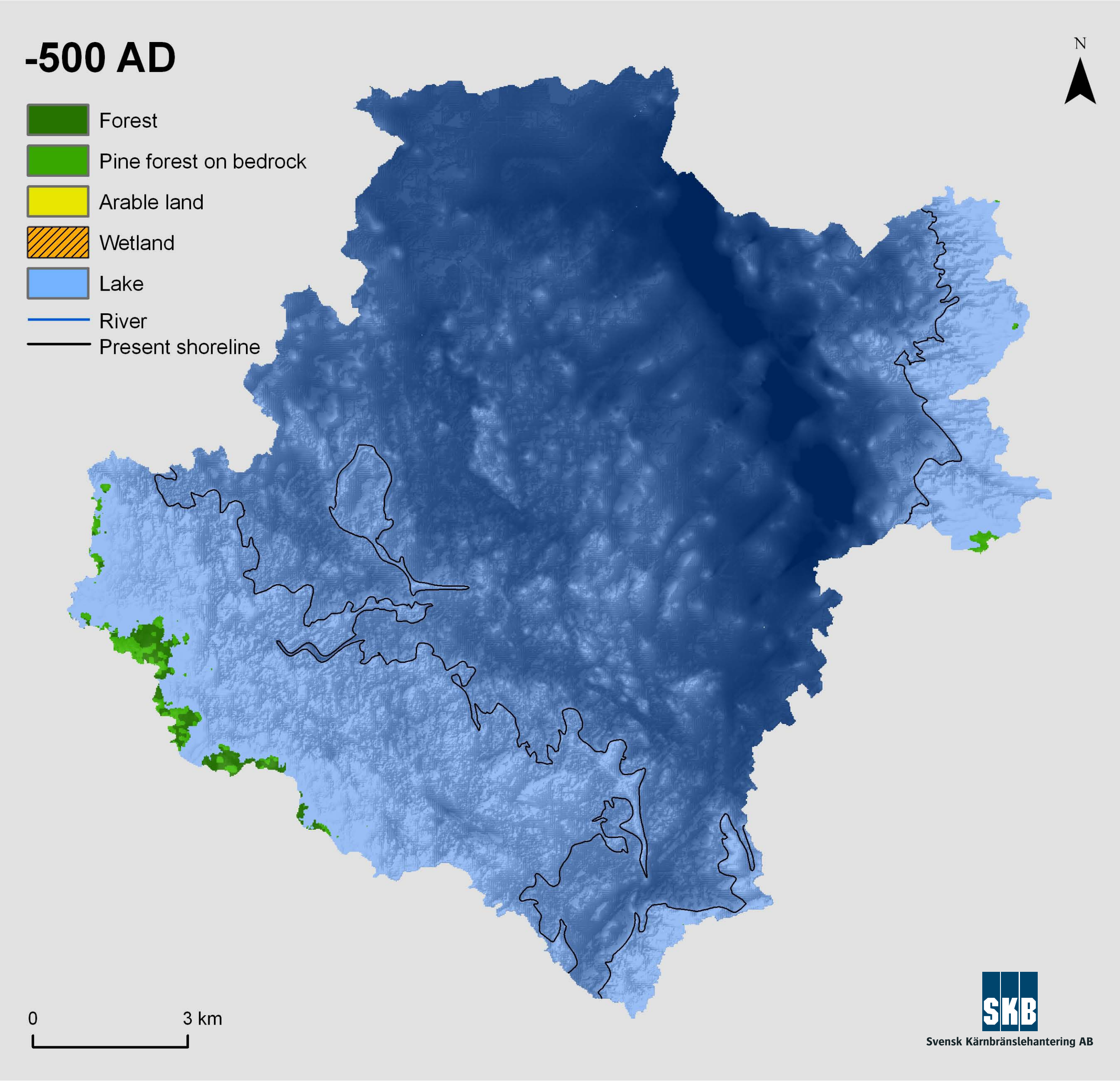
-500 AD
Forest
Pine forest on bedrock
Arable land
Wetland
Lake
River
Present shoreline
N
0
3 km
SKB
Svensk Kärnbränslehantering AB

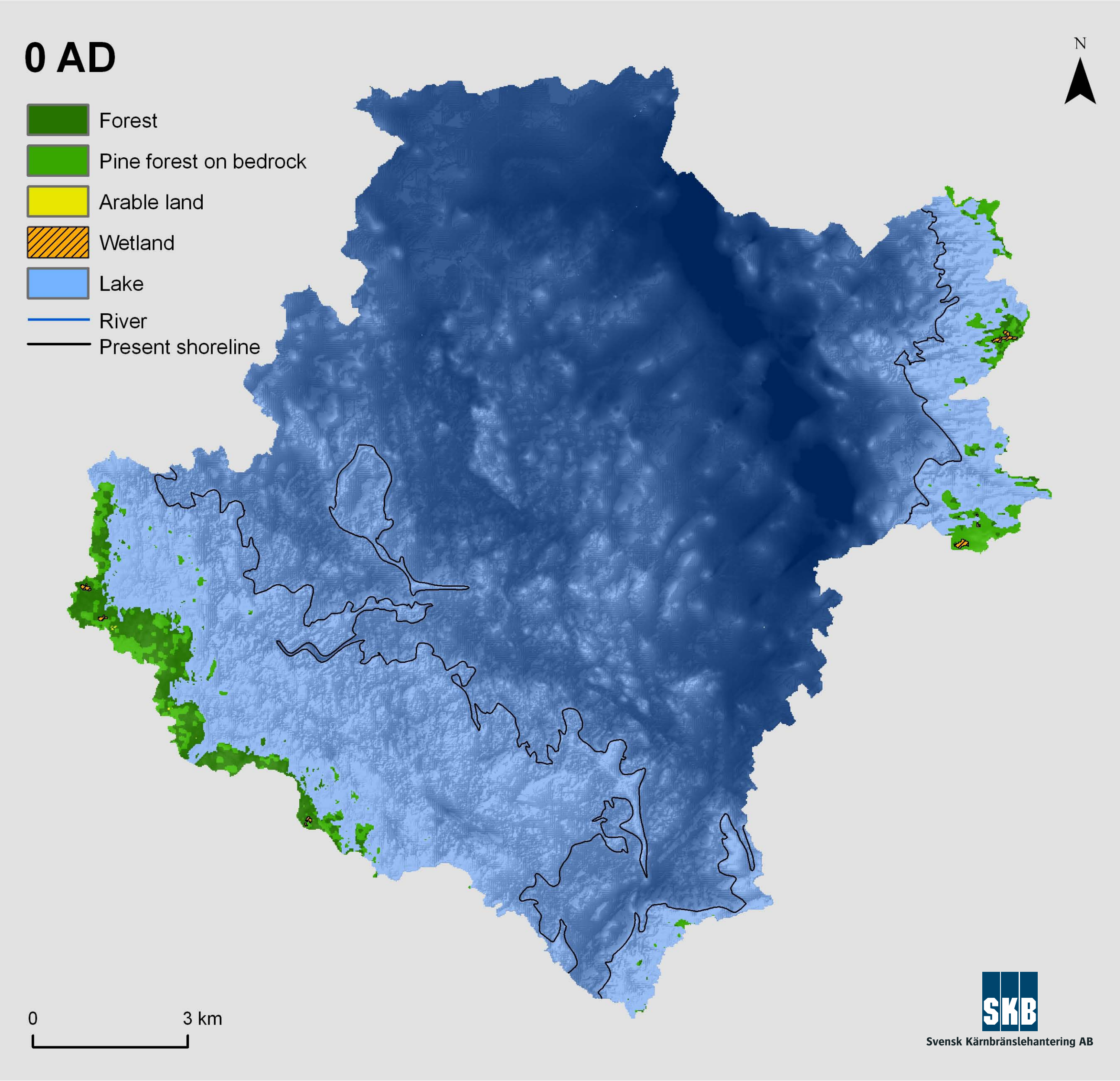
0 AD
Forest
Pine forest on bedrock
Arable land
Wetland
Lake
River
Present shoreline
N
0
3 km
SKB
Svensk Kärnbränslehantering AB

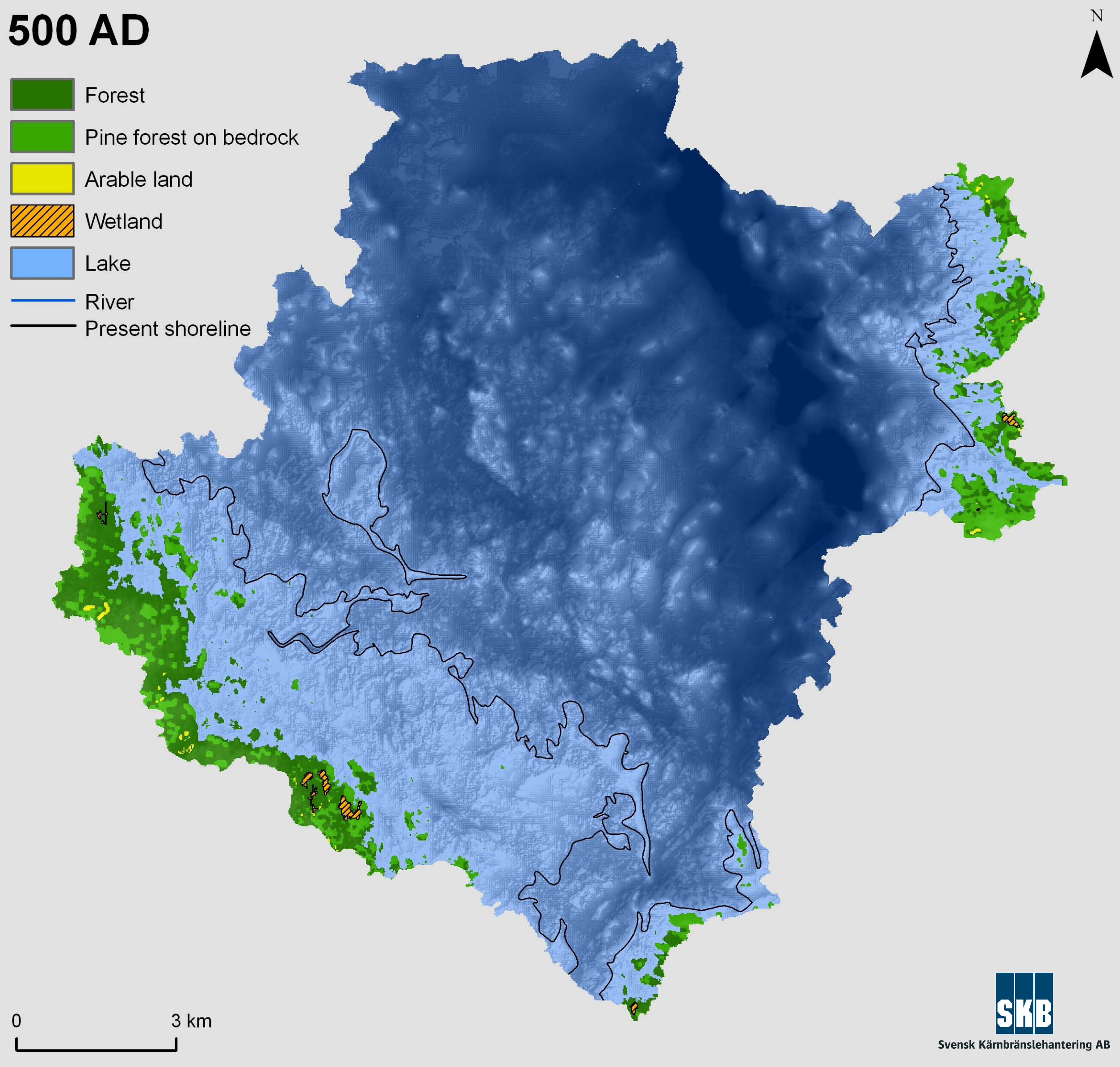
500 AD
Forest
Pine forest on bedrock
Arable land
Wetland
Lake
River
Present shoreline
N
0
3 km
SKB
Svensk Kärnbränslehantering AB

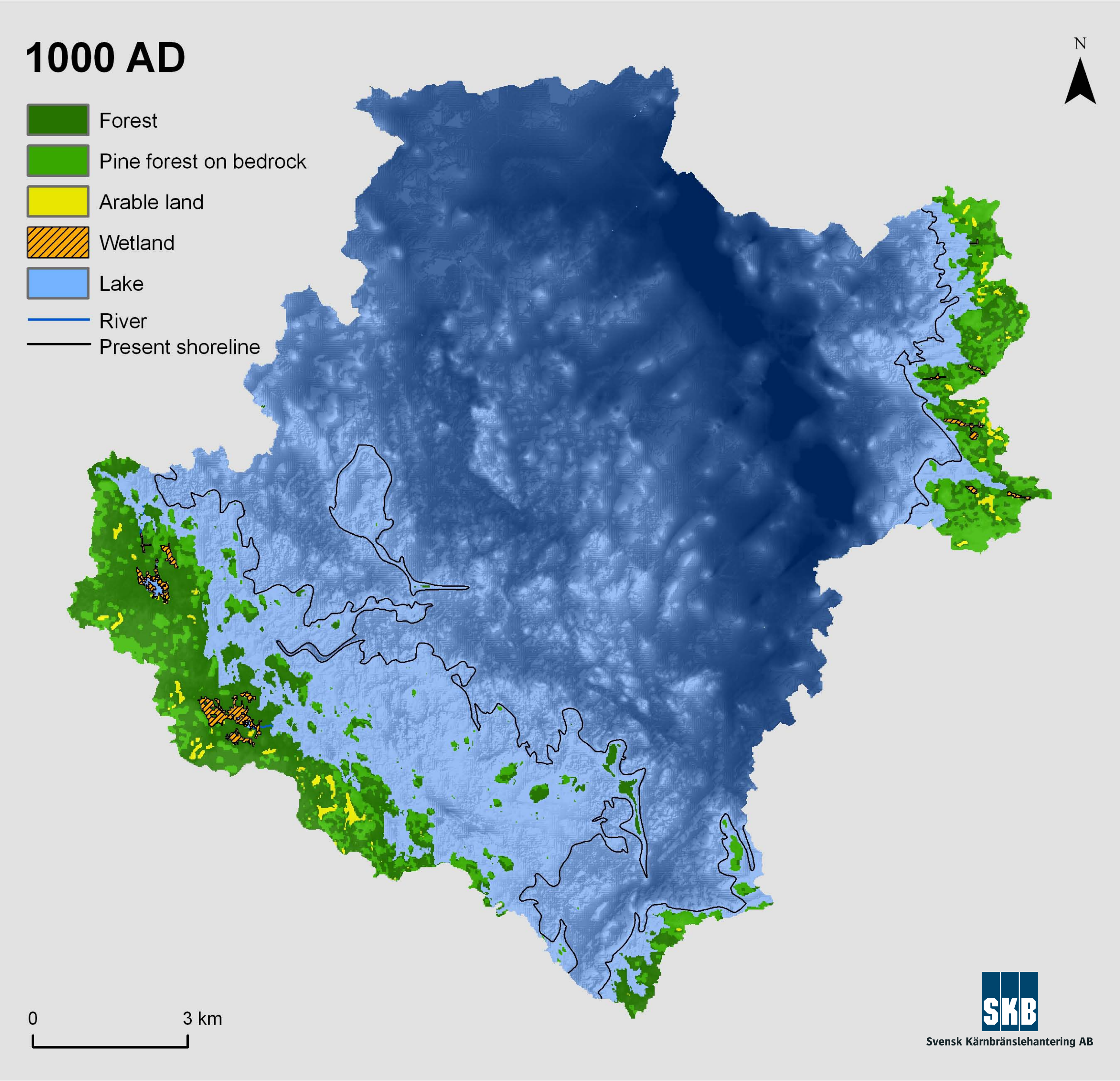
1000 AD
Forest
Pine forest on bedrock
Arable land
Wetland
Lake
River
Present shoreline
N
0
3 km
SKB
Svensk Kärnbränslehantering AB

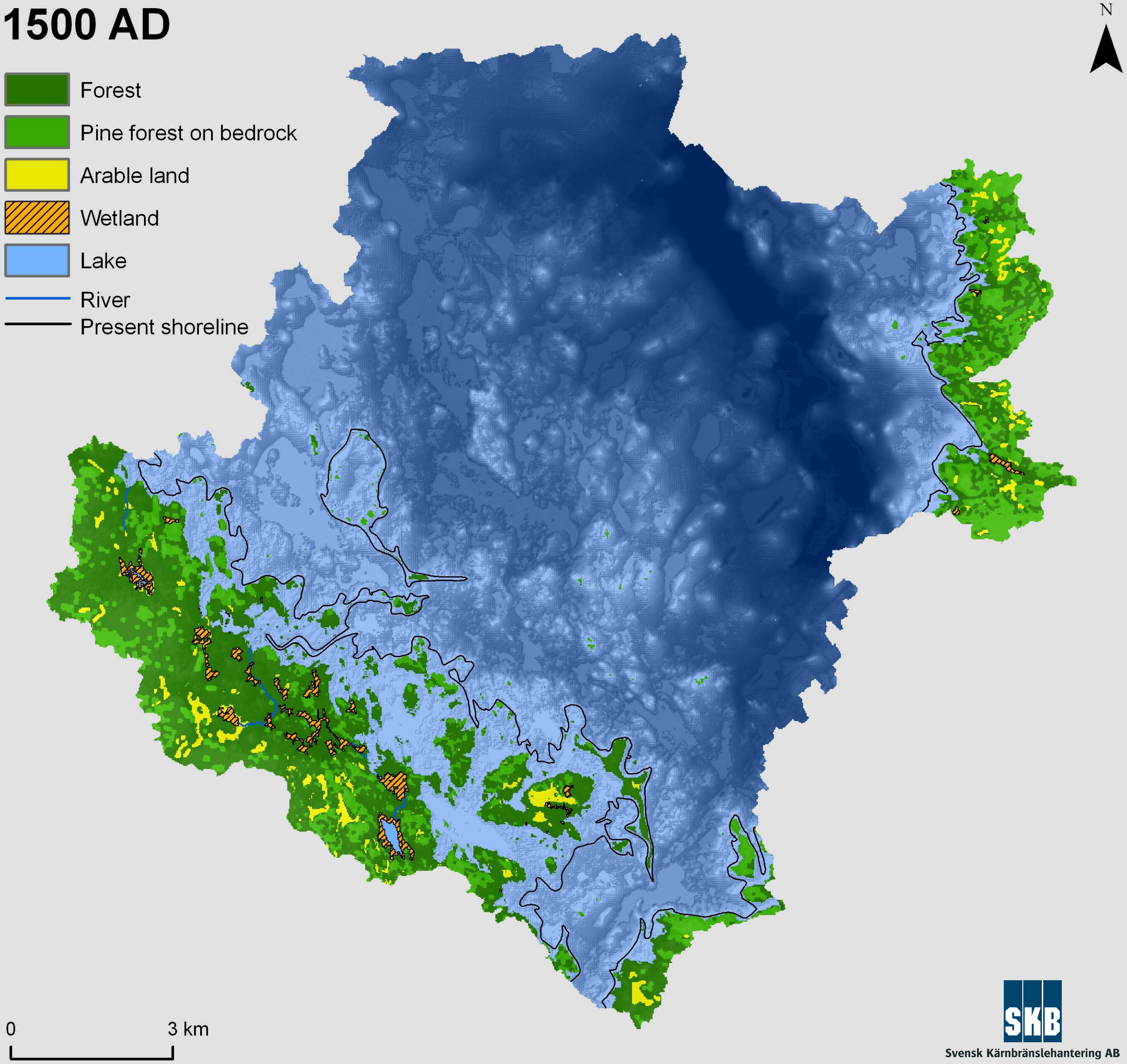
1500 AD
Forest
Pine forest on bedrock
Arable land
Wetland
Lake
River
Present shoreline
N
0
3 km
SKB
Svensk Kärnbränslehantering AB

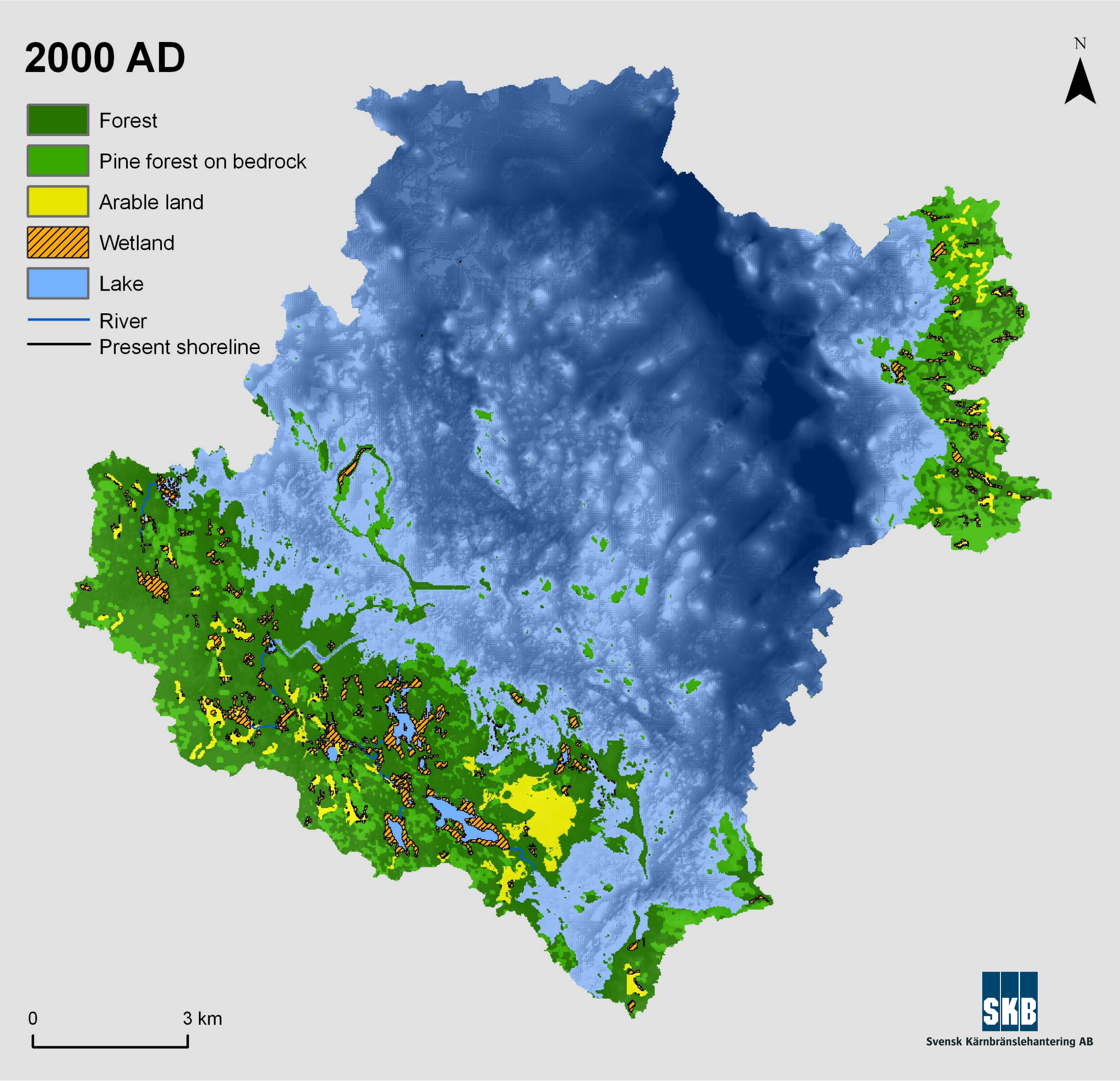
2000 AD
Forest
Pine forest on bedrock
Arable land
Wetland
Lake
River
Present shoreline
N
0
3 km
SKB
Svensk Kärnbränslehantering AB

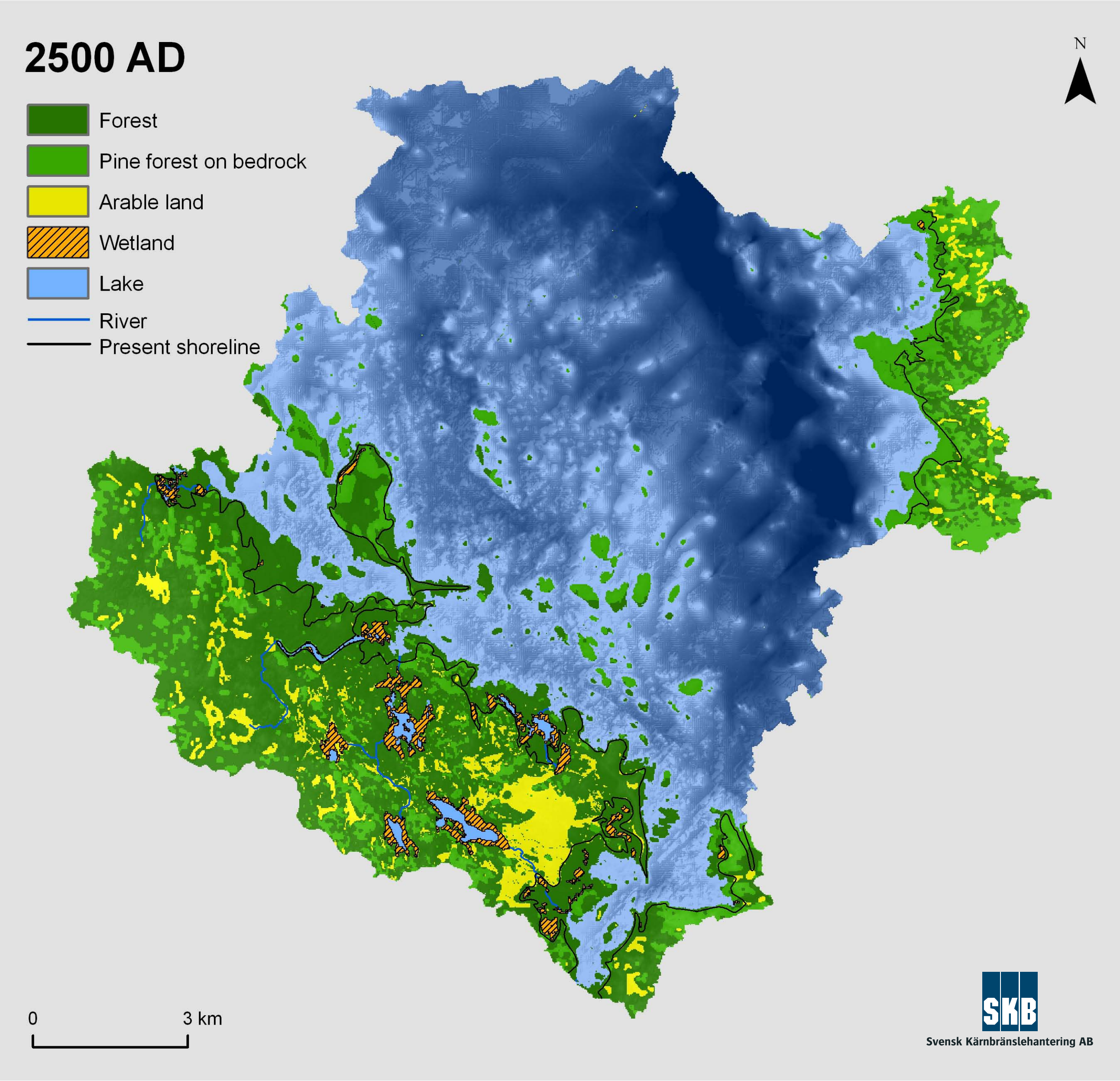
2500 AD
Forest
Pine forest on bedrock
Arable land
Wetland
Lake
River
Present shoreline
N
0
3 km
SKB
Svensk Kärnbränslehantering AB

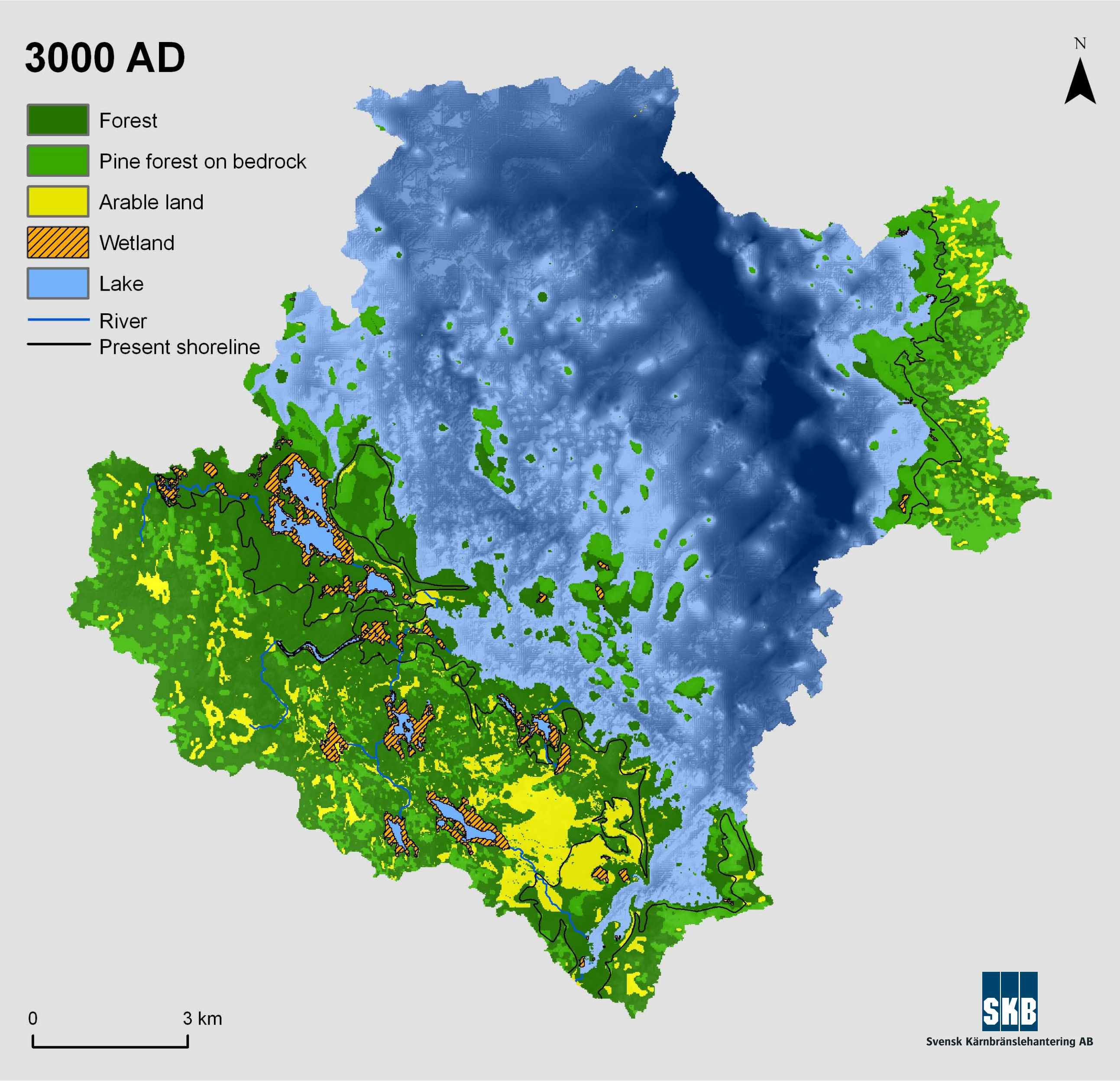
3000 AD
Forest
Pine forest on bedrock
Arable land
Wetland
Lake
River
Present shoreline
N
0
3 km
SKB
Svensk Kärnbränslehantering AB

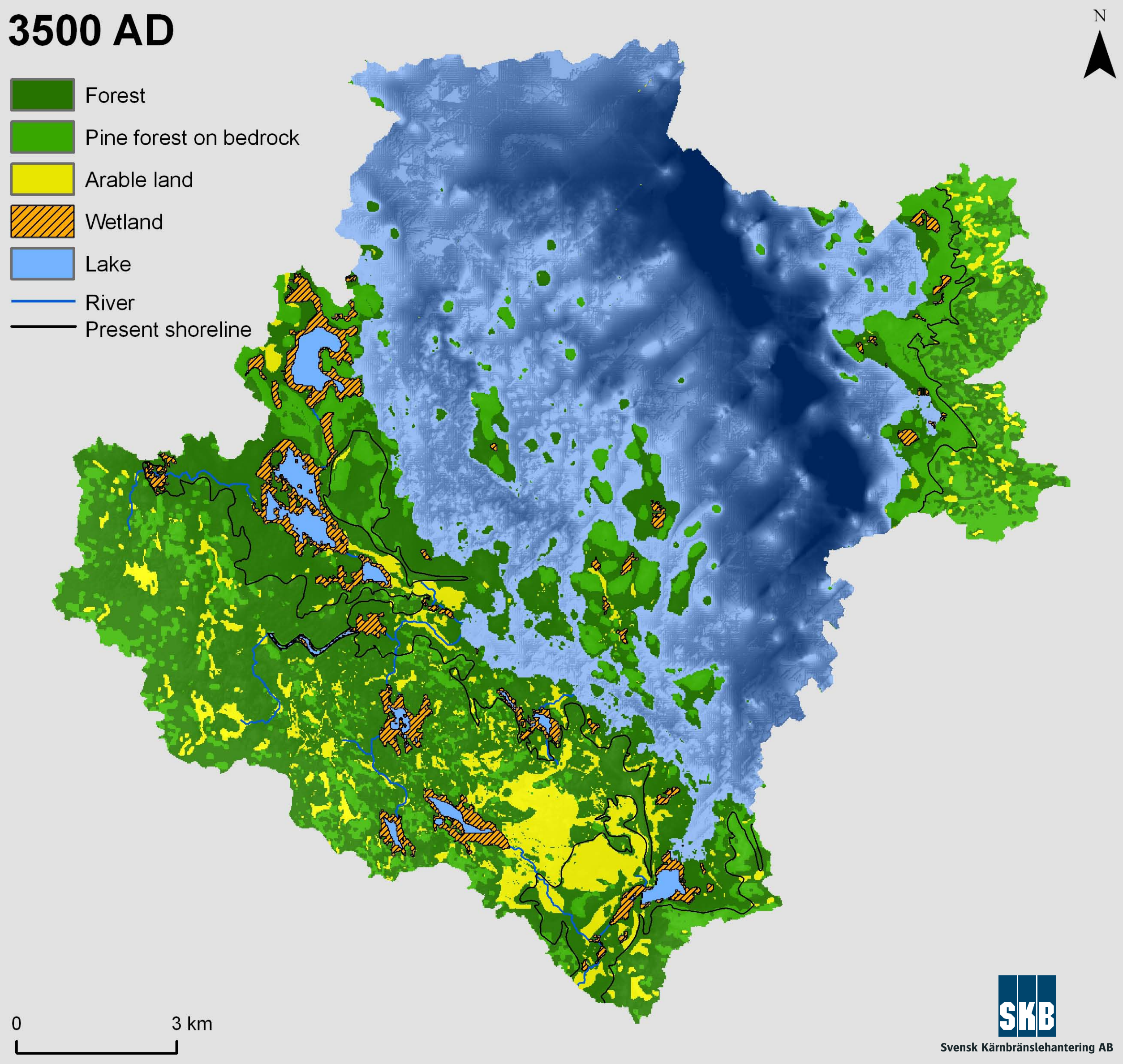

3500 AD
Forest
Pine forest on bedrock
Arable land
Wetland
Lake
River
Present shoreline
N
0
3 km
SKB
Svensk Kärnbränslehantering AB

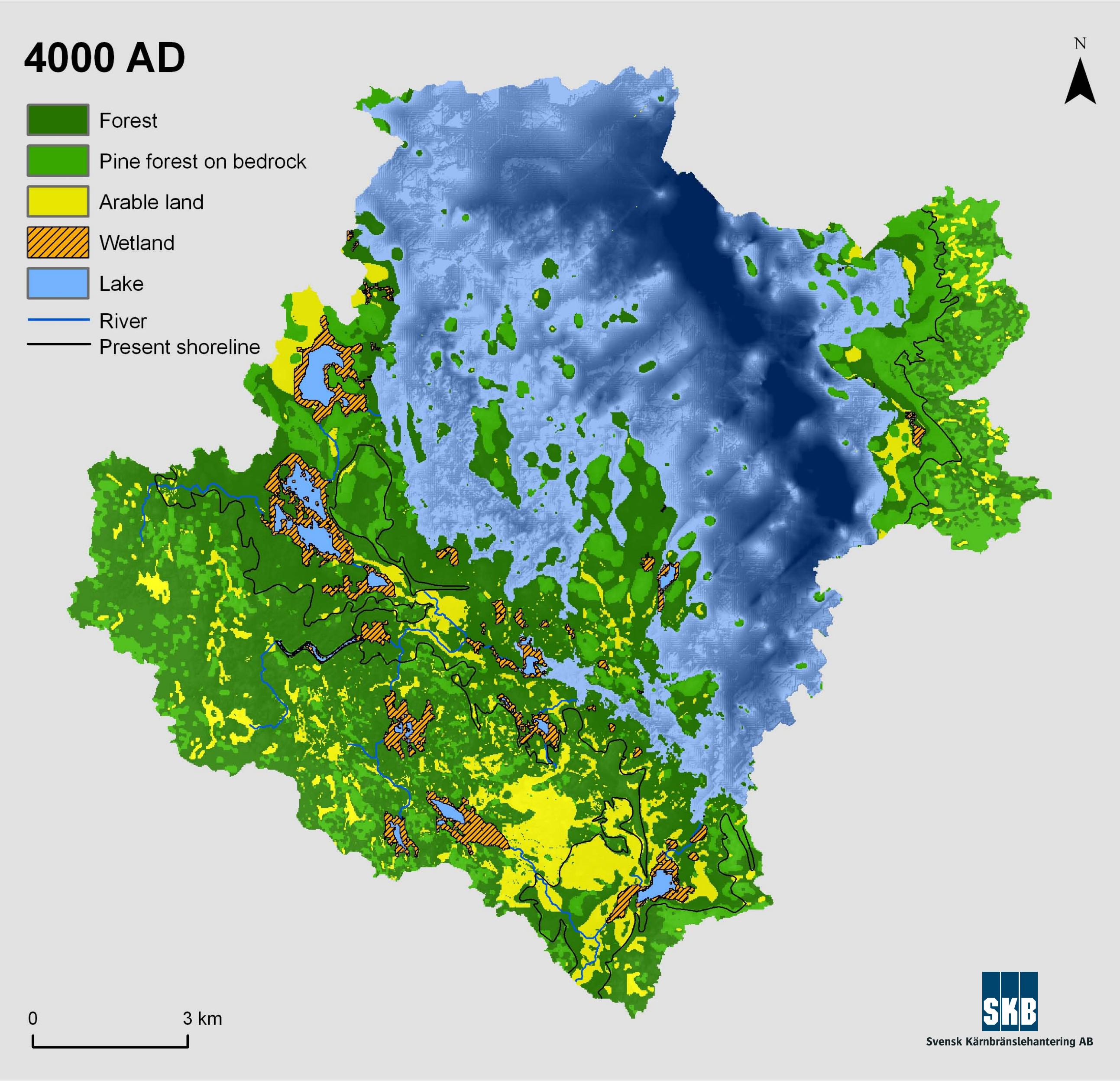
4000 AD
Forest
Pine forest on bedrock
Arable land
Wetland
Lake
River
Present shoreline
N
0
3 km
SKB
Svensk Kärnbränslehantering AB

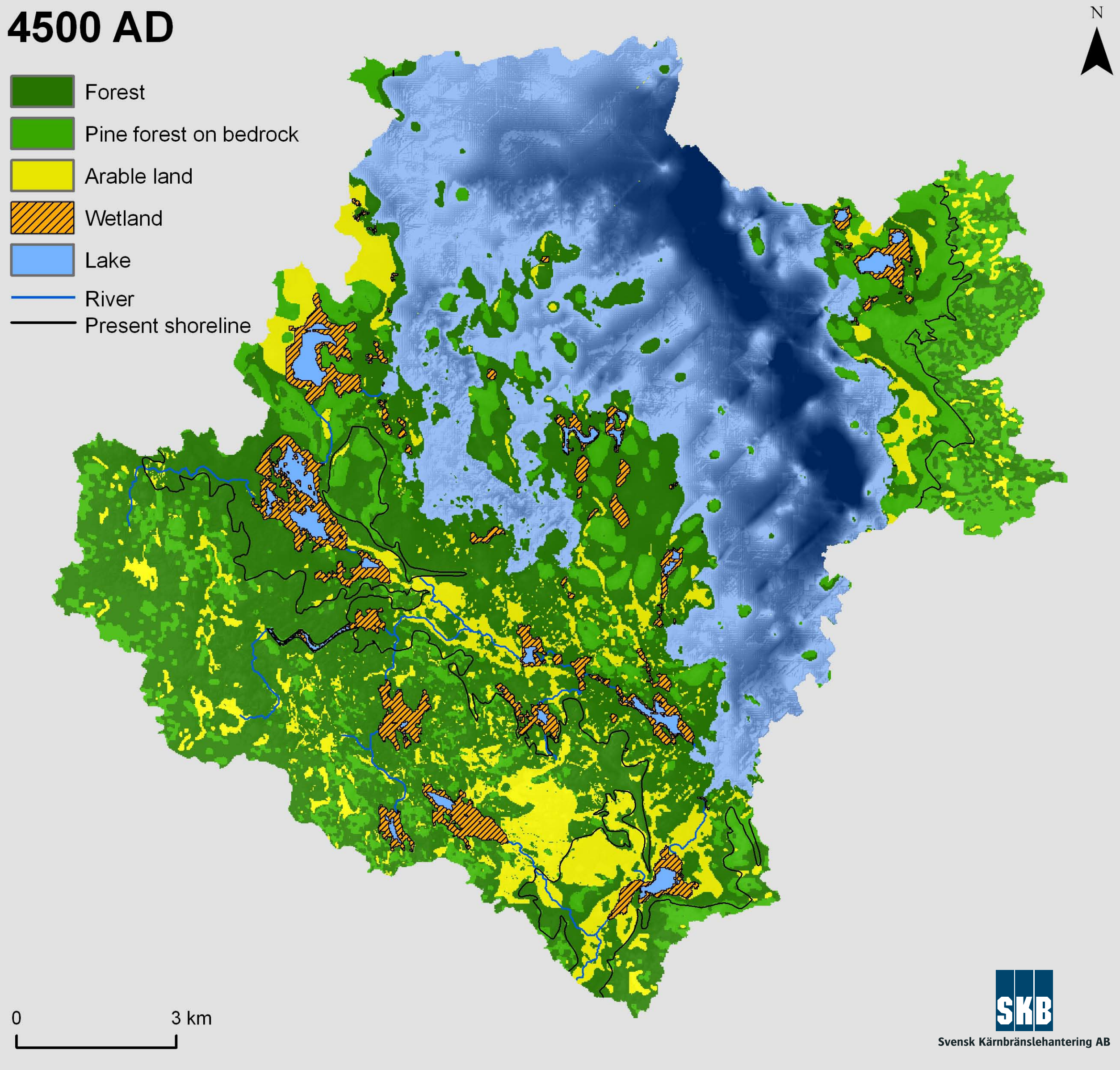
4500 AD
Forest
Pine forest on bedrock
Arable land
Wetland
Lake
River
Present shoreline
N
0
3 km
SKB
Svensk Kärnbränslehantering AB

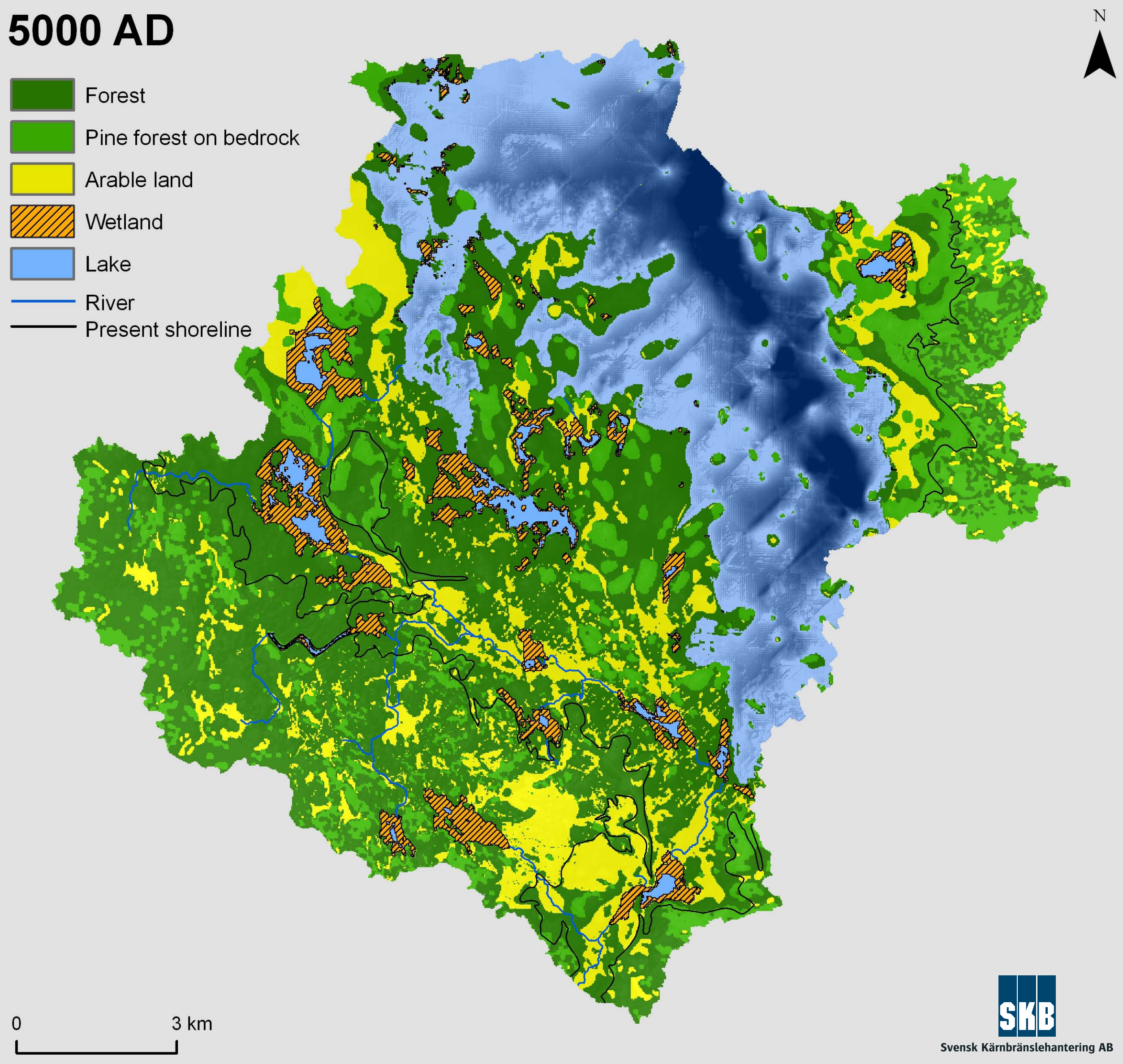
5000 AD
Forest
Pine forest on bedrock
Arable land
Wetland
Lake
River
Present shoreline
N
0
3 km
SKB
Svensk Kärnbränslehantering AB

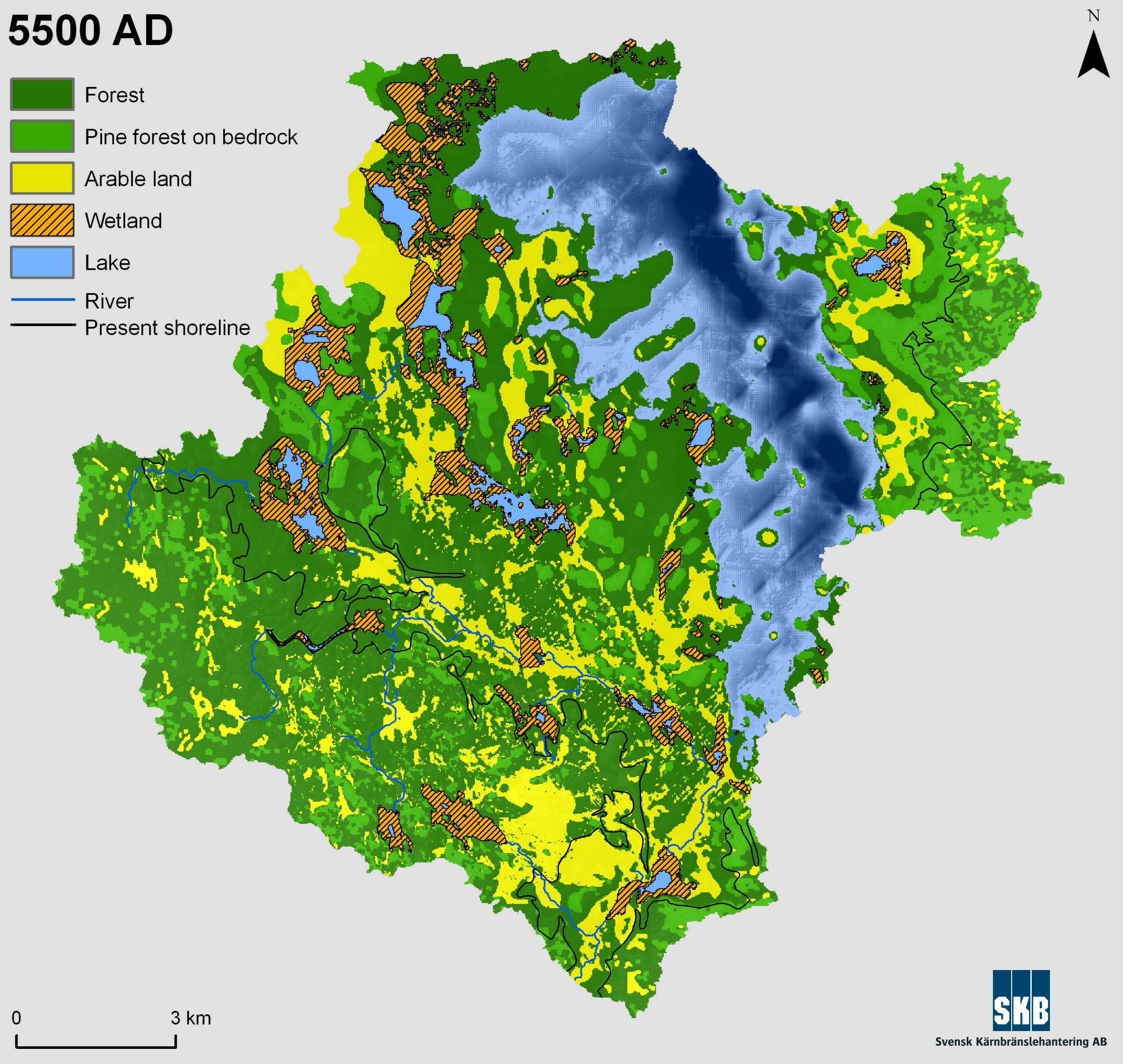
5500 AD
Forest
Pine forest on bedrock
Arable land
Wetland
Lake
River
Present shoreline
N
0
3 km
SKB
Svensk Kärnbränslehantering AB

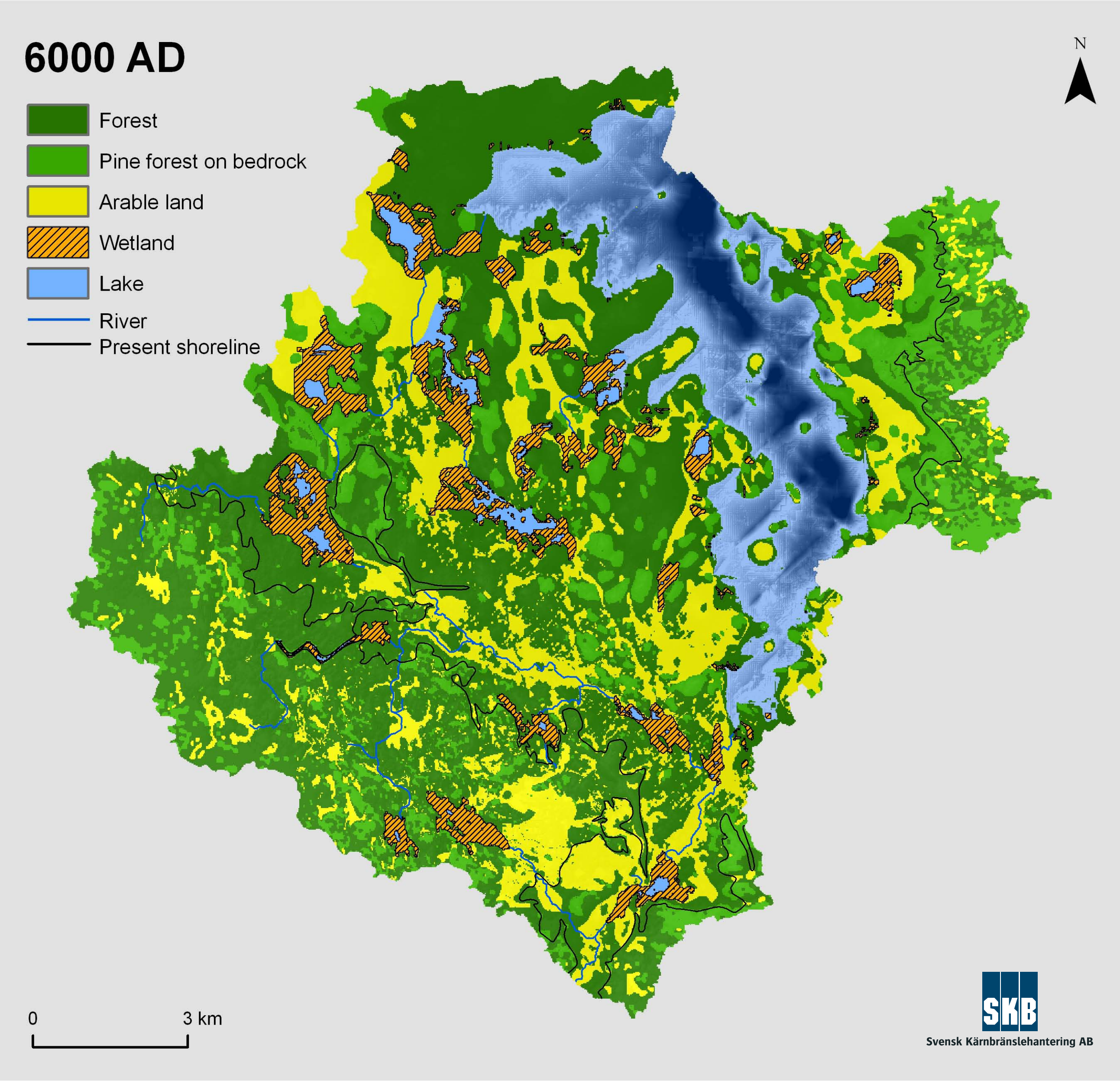
6000 AD
Forest
Pine forest on bedrock
Arable land
Wetland
Lake
River
Present shoreline
N
0
3 km
SKB
Svensk Kärnbränslehantering AB

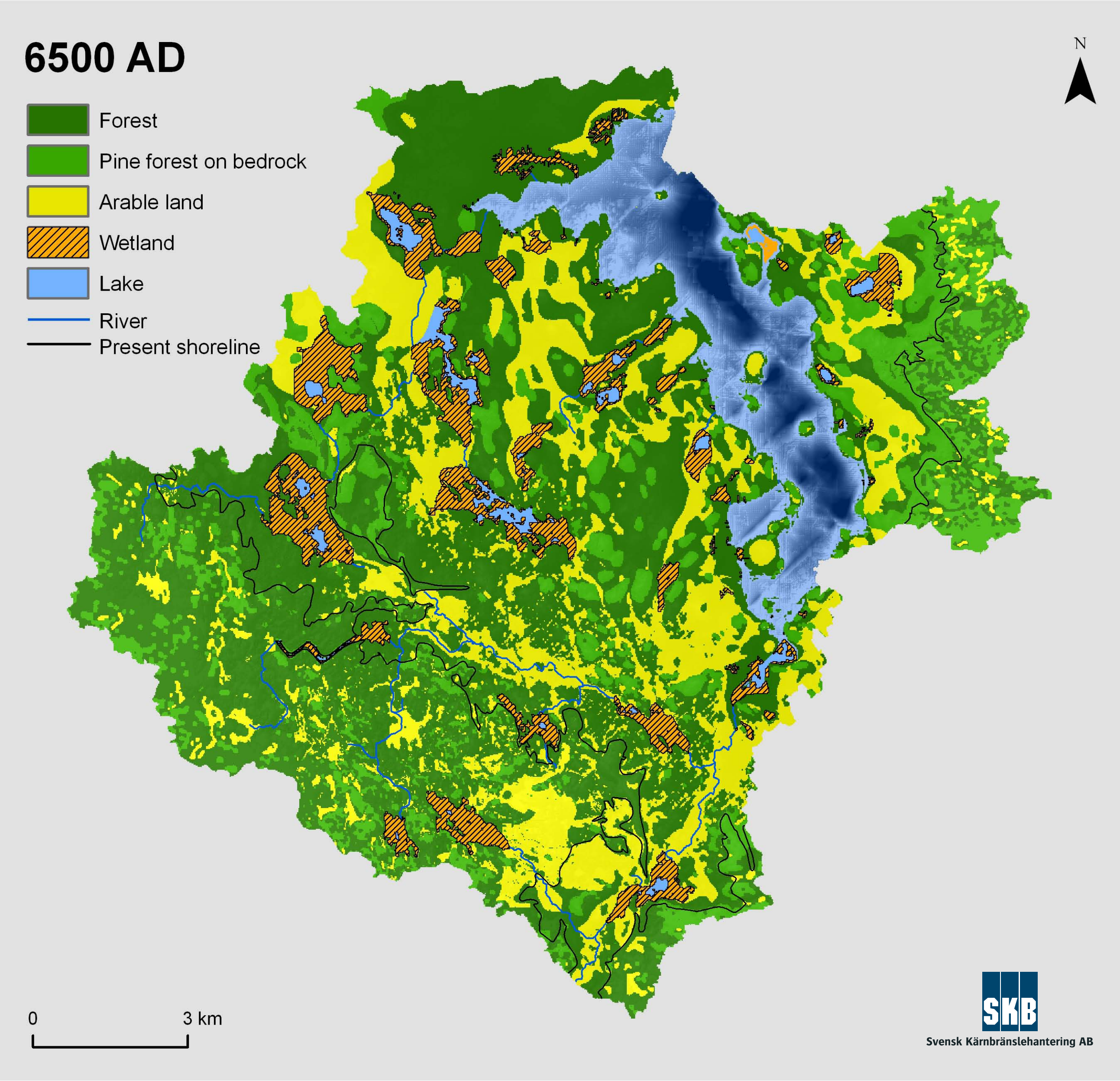
6500 AD
Forest
Pine forest on bedrock
Arable land
Wetland
Lake
River
Present shoreline
N
0
3 km
SKB
Svensk Kärnbränslehantering AB

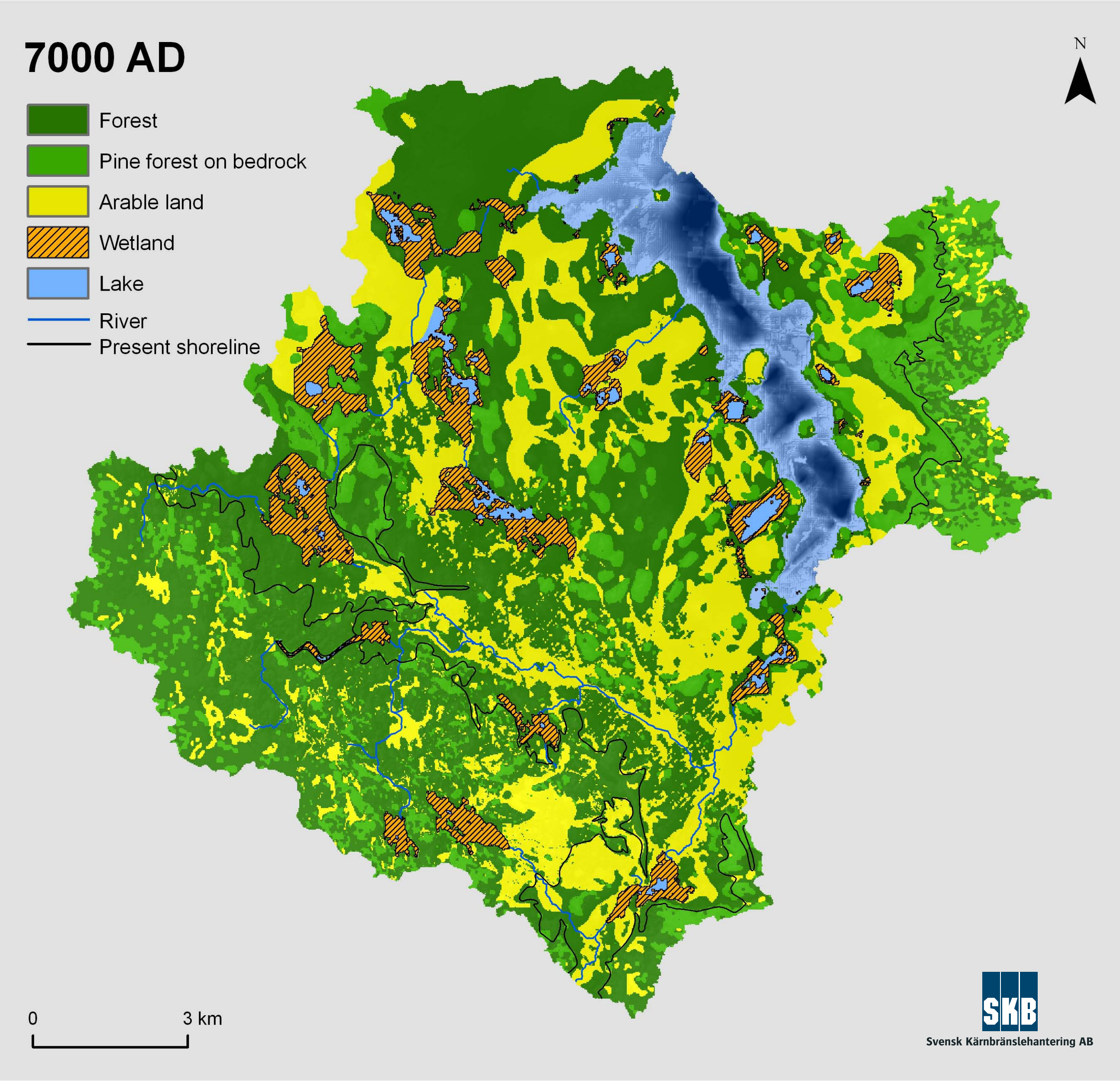

7000 AD
Forest
Pine forest on bedrock
Arable land
Wetland
Lake
River
Present shoreline
N
0
3 km
SKB
Svensk Kärnbränslehantering AB

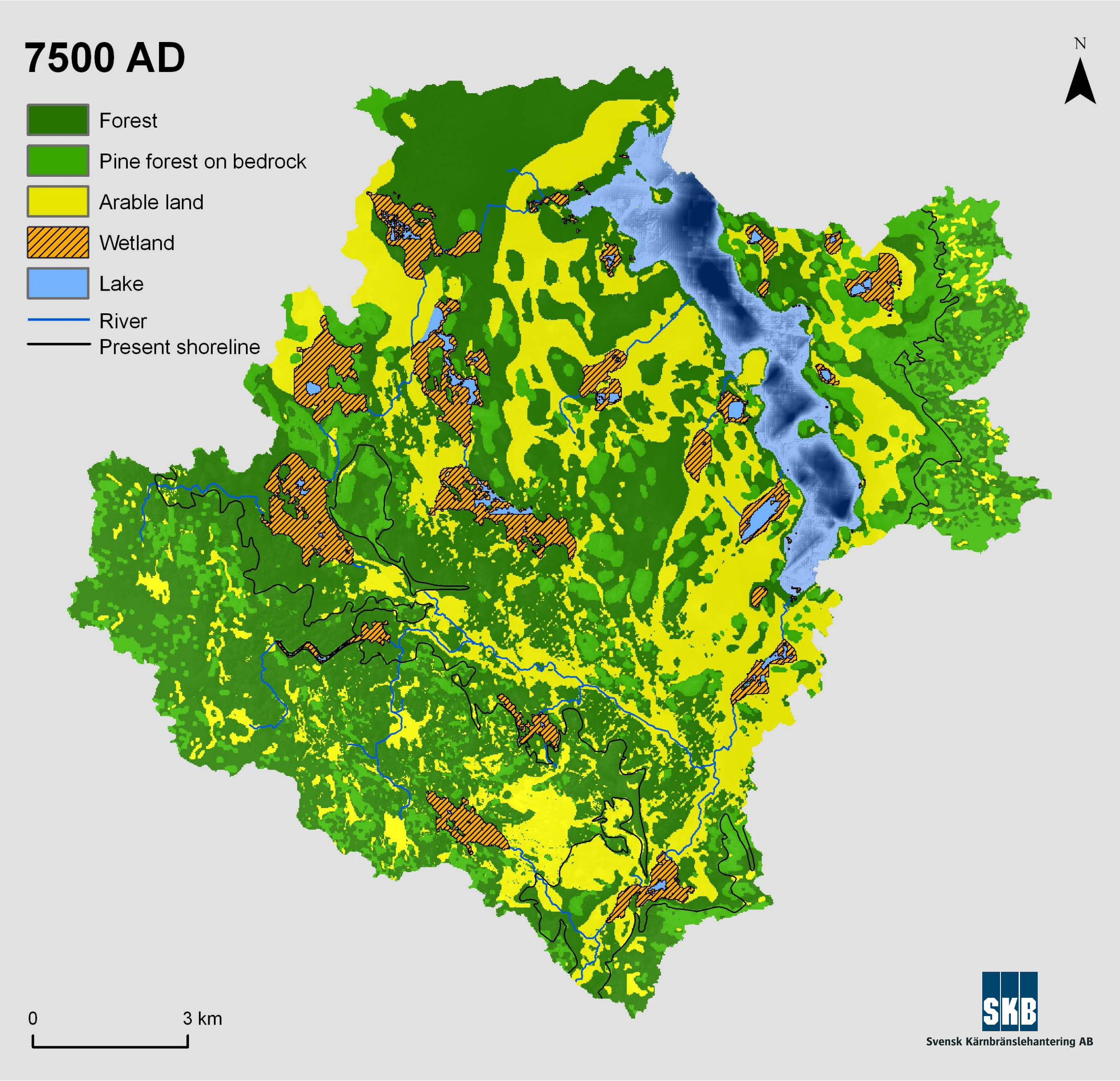
7500 AD
Forest
Pine forest on bedrock
Arable land
Wetland
Lake
River
Present shoreline
N
0
3 km
SKB
Svensk Kärnbränslehantering AB

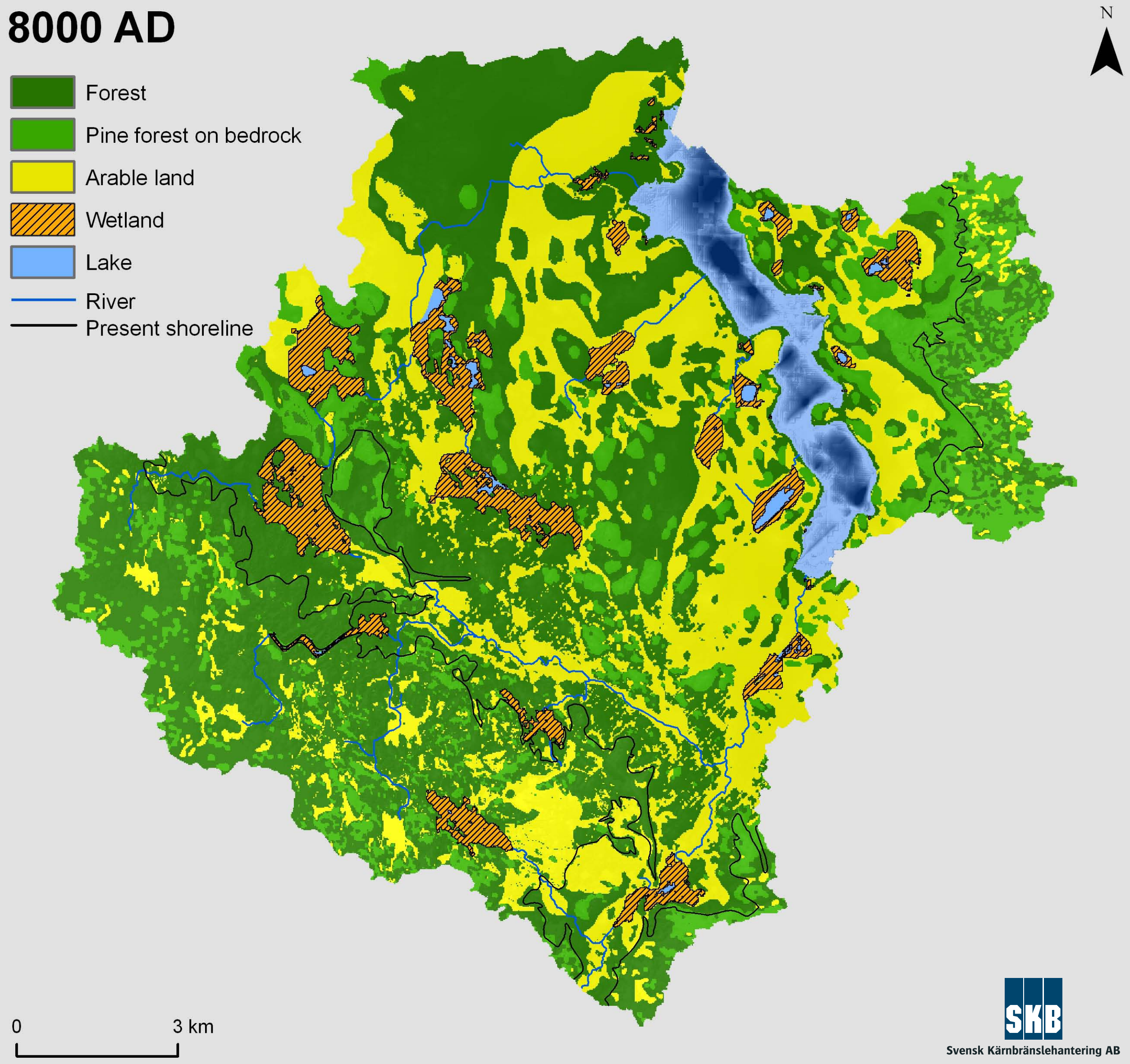
8000 AD
Forest
Pine forest on bedrock
Arable land
Wetland
Lake
River
Present shoreline
N
0
3 km
Svensk Kärnbränslehantering AB

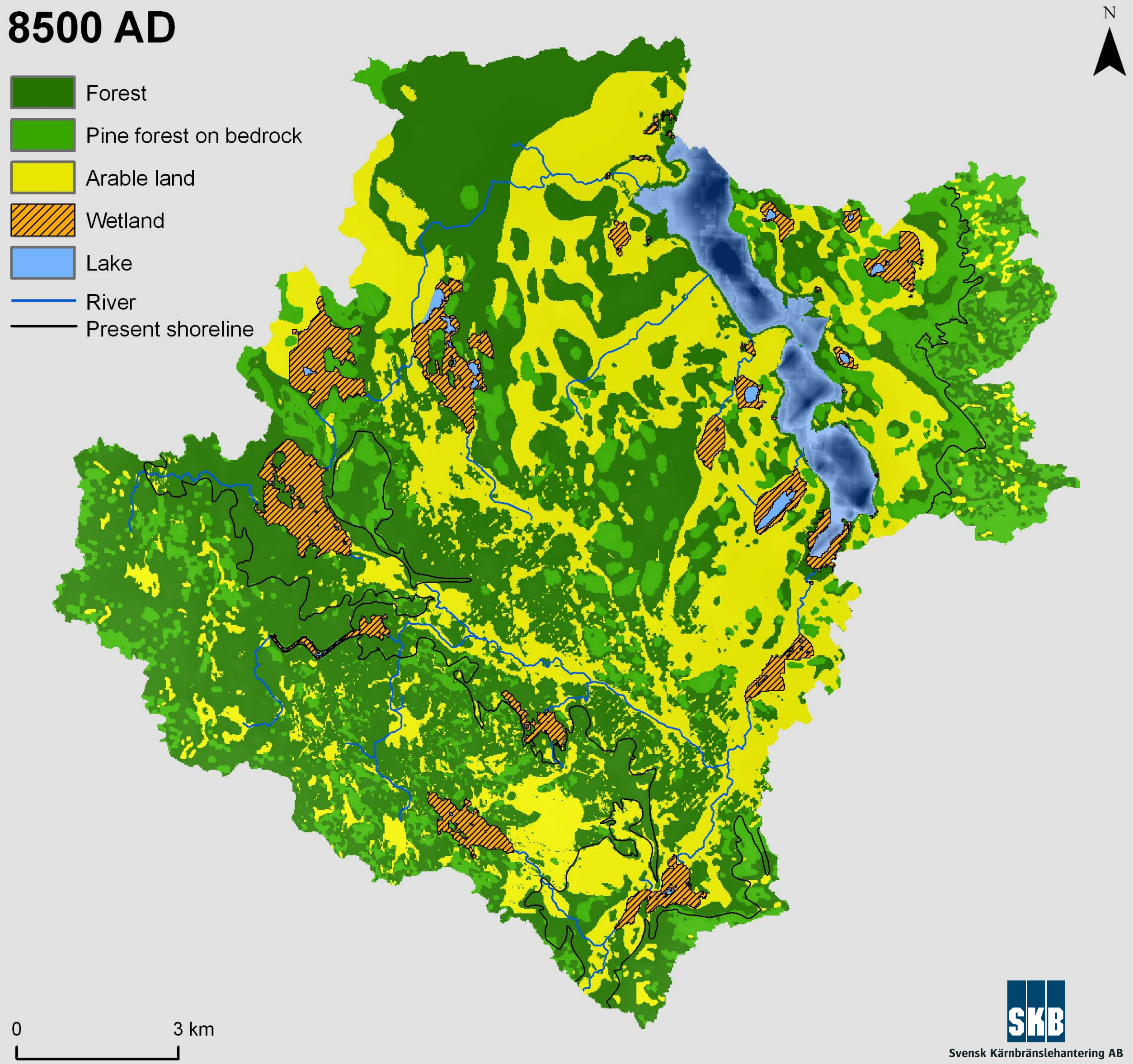
8500 AD
Forest
Pine forest on bedrock
Arable land
Wetland
Lake
River
Present shoreline
N
0
3 km
SKB
Svensk Kärnbränslehantering AB

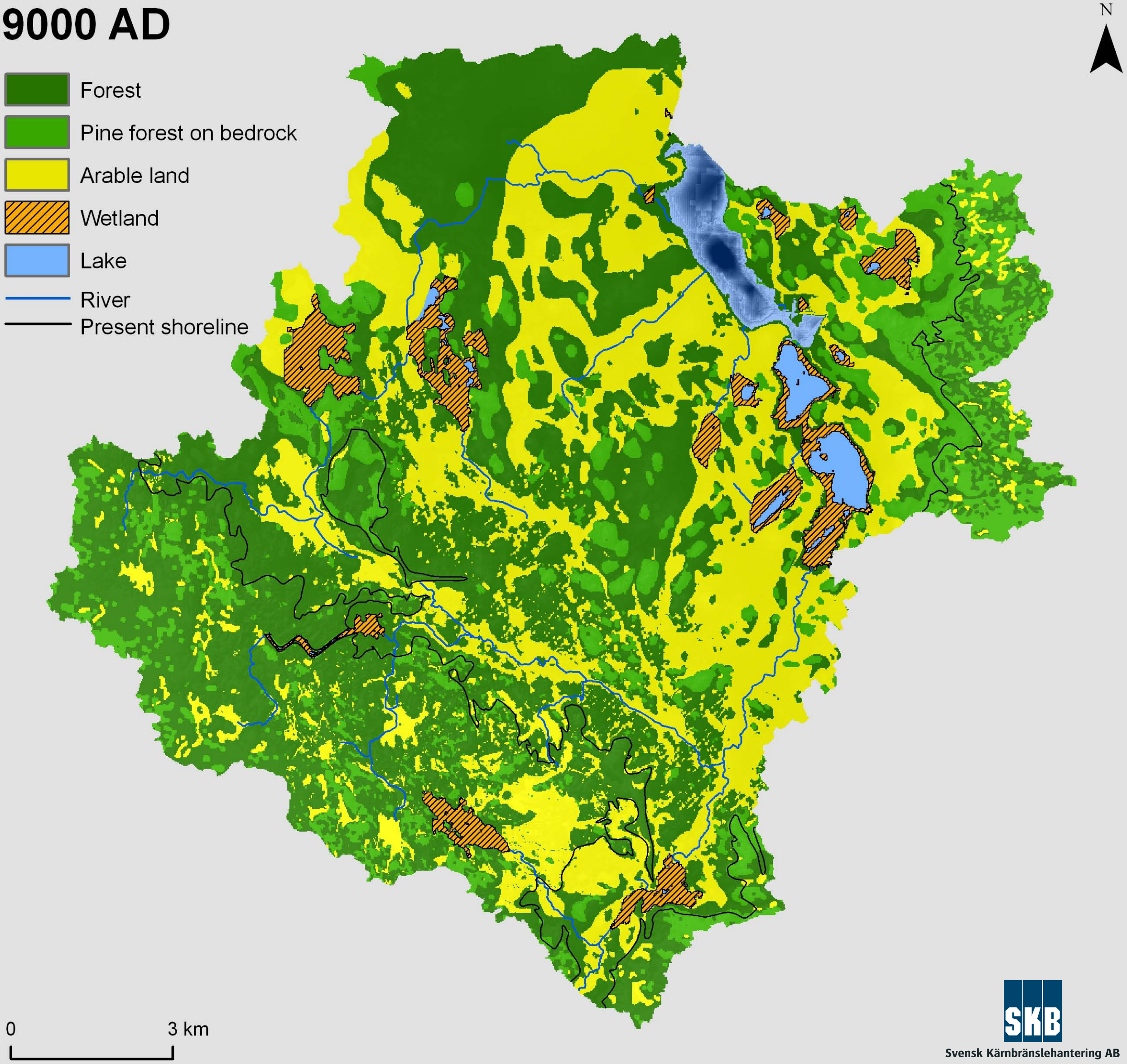

9000 AD
Forest
Pine forest on bedrock
Arable land
Wetland
Lake
River
Present shoreline
N
0
3 km
SKB
Svensk Kärnbränslehantering AB

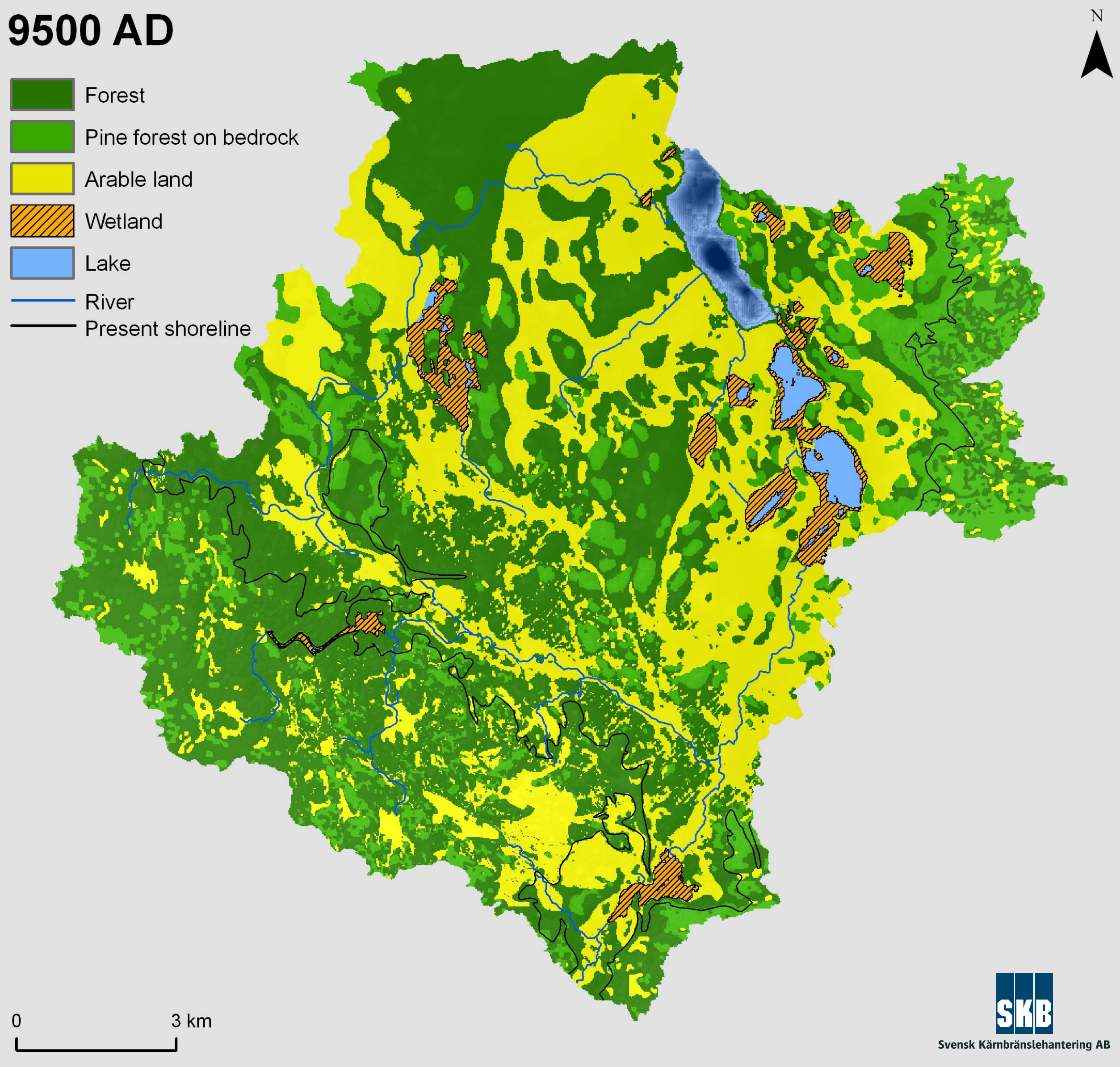

9500 AD
Forest
Pine forest on bedrock
Arable land
Wetland
Lake
River
Present shoreline
N
0
3 km
SKB
Svensk Kärnbränslehantering AB

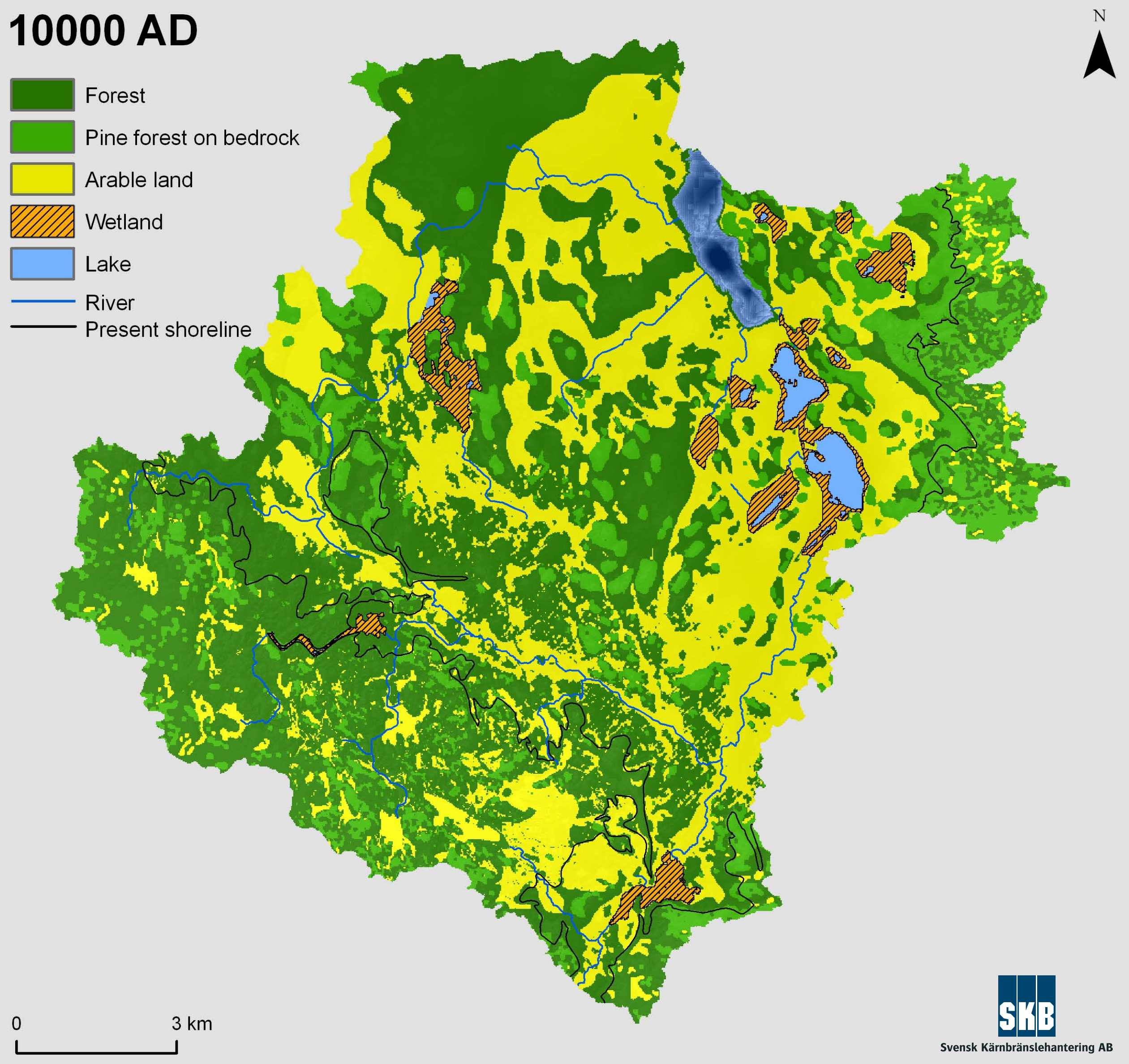

10000 AD
Forest
Pine forest on bedrock
Arable land
Wetland
Lake
River
Present shoreline
N
0
3 km
SKB
Svensk Kärnbränslehantering AB

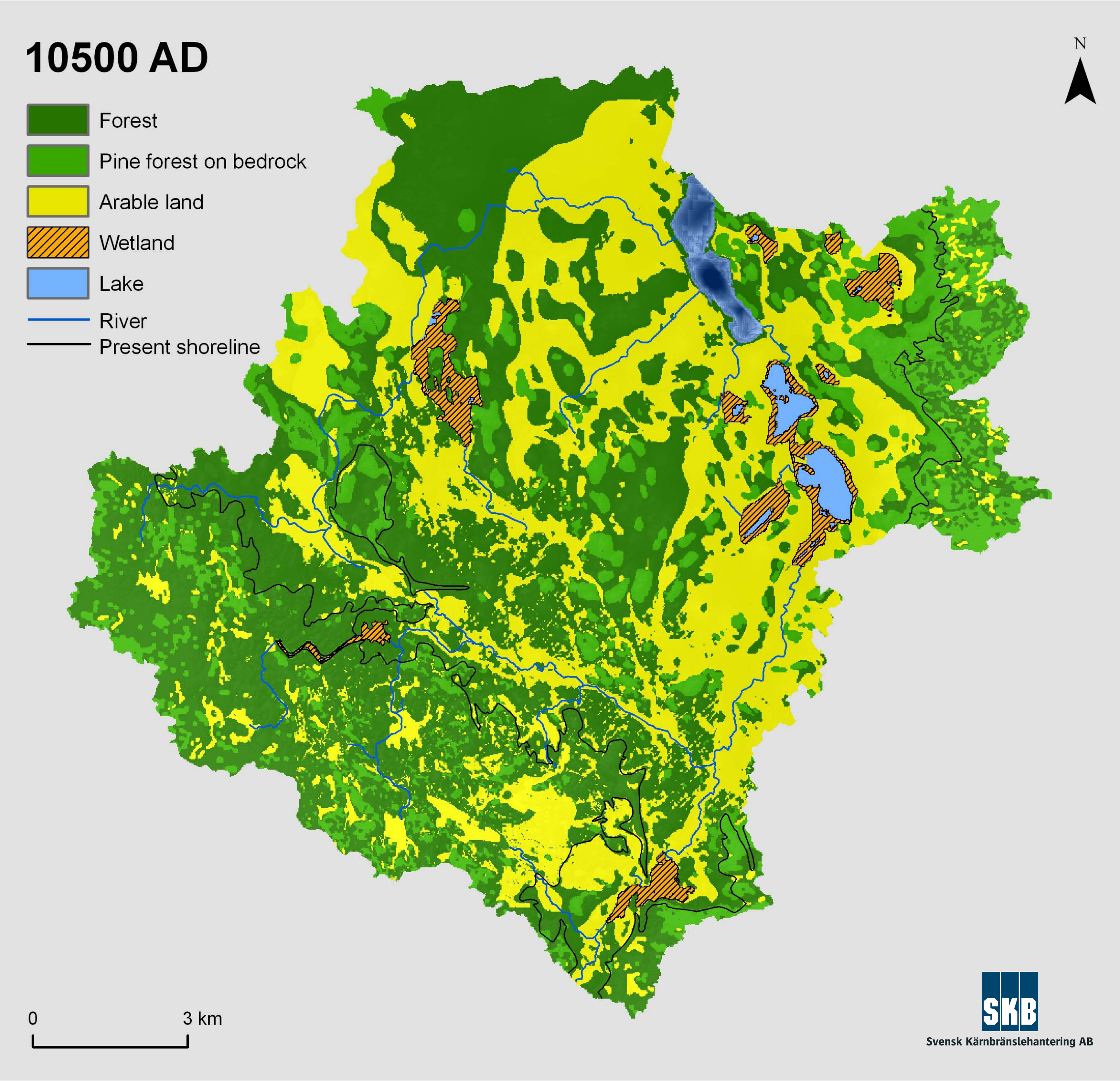
10500 AD
Forest
Pine forest on bedrock
Arable land
Wetland
Lake
River
Present shoreline
N
0
3 km
SKB
Svensk Kärnbränslehantering AB

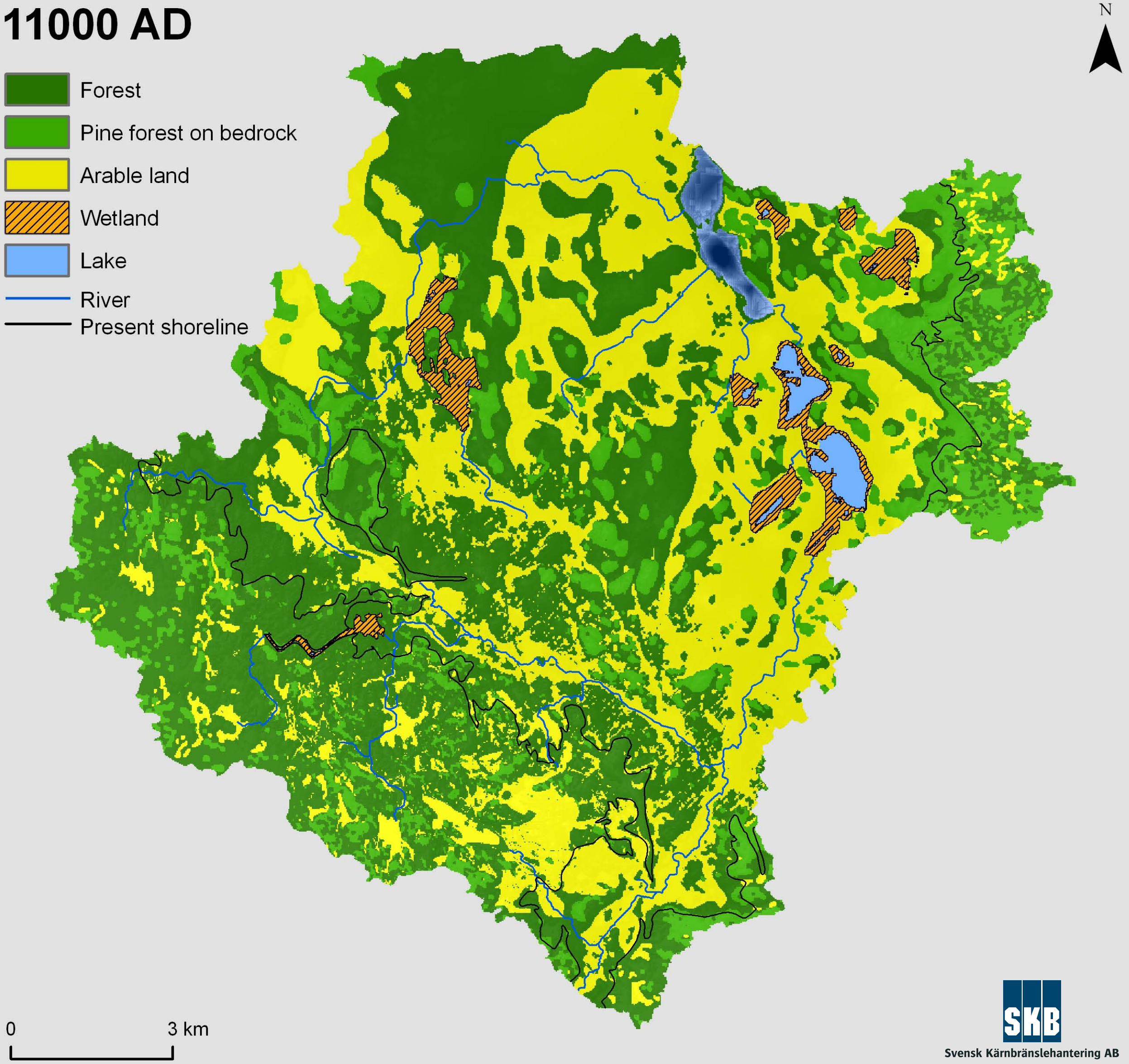
11000 AD
Forest
Pine forest on bedrock
Arable land
Wetland
Lake
River
Present shoreline
N
0
3 km
SKB
Svensk Kärnbränslehantering AB

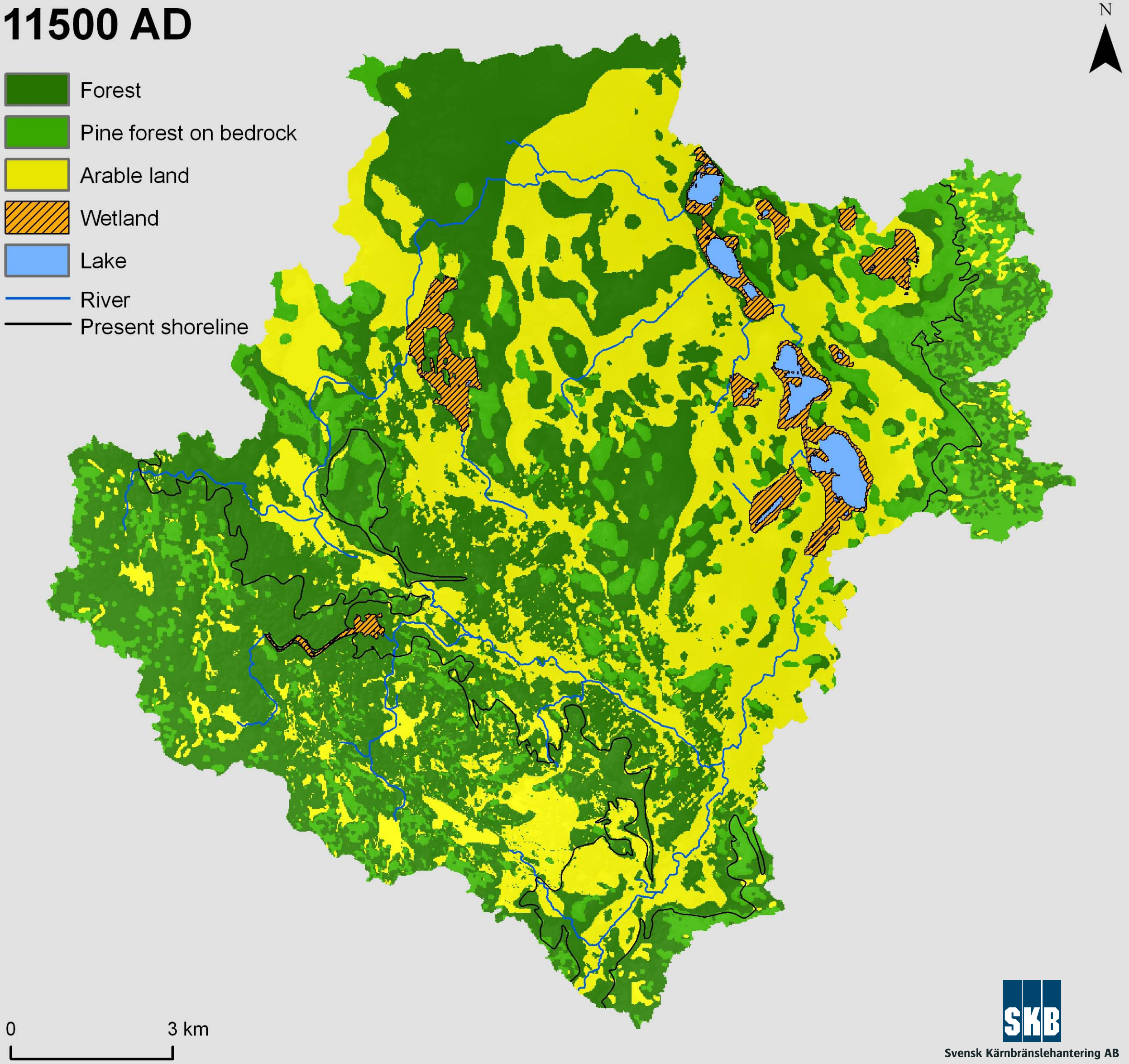
11500 AD
Forest
Pine forest on bedrock
Arable land
Wetland
Lake
River
Present shoreline
N
0
3 km
Svensk Kärnbränslehantering AB

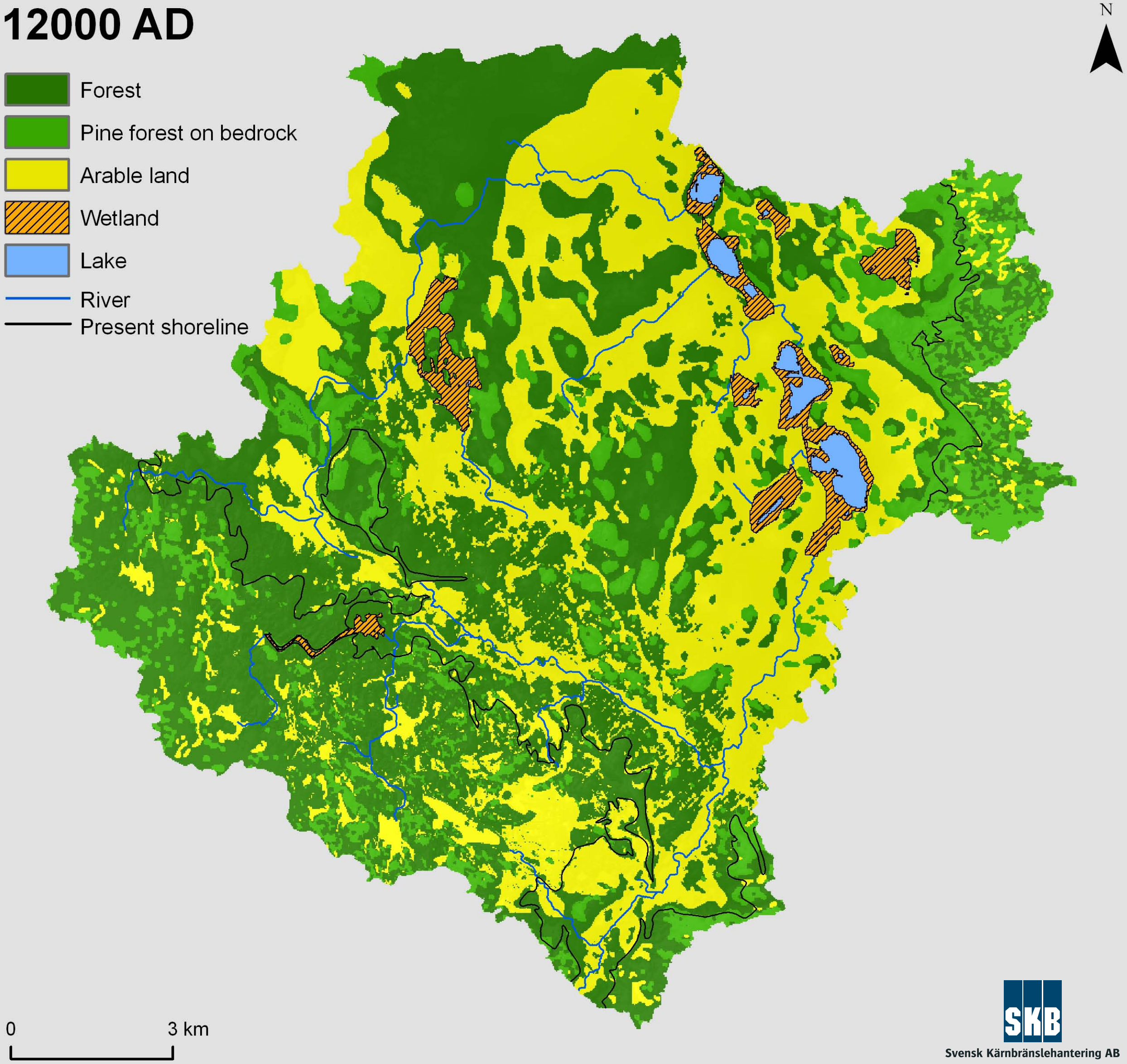
12000 AD
Forest
Pine forest on bedrock
Arable land
Wetland
Lake
River
Present shoreline
N
0
3 km
SKB
Svensk Kärnbränslehantering AB

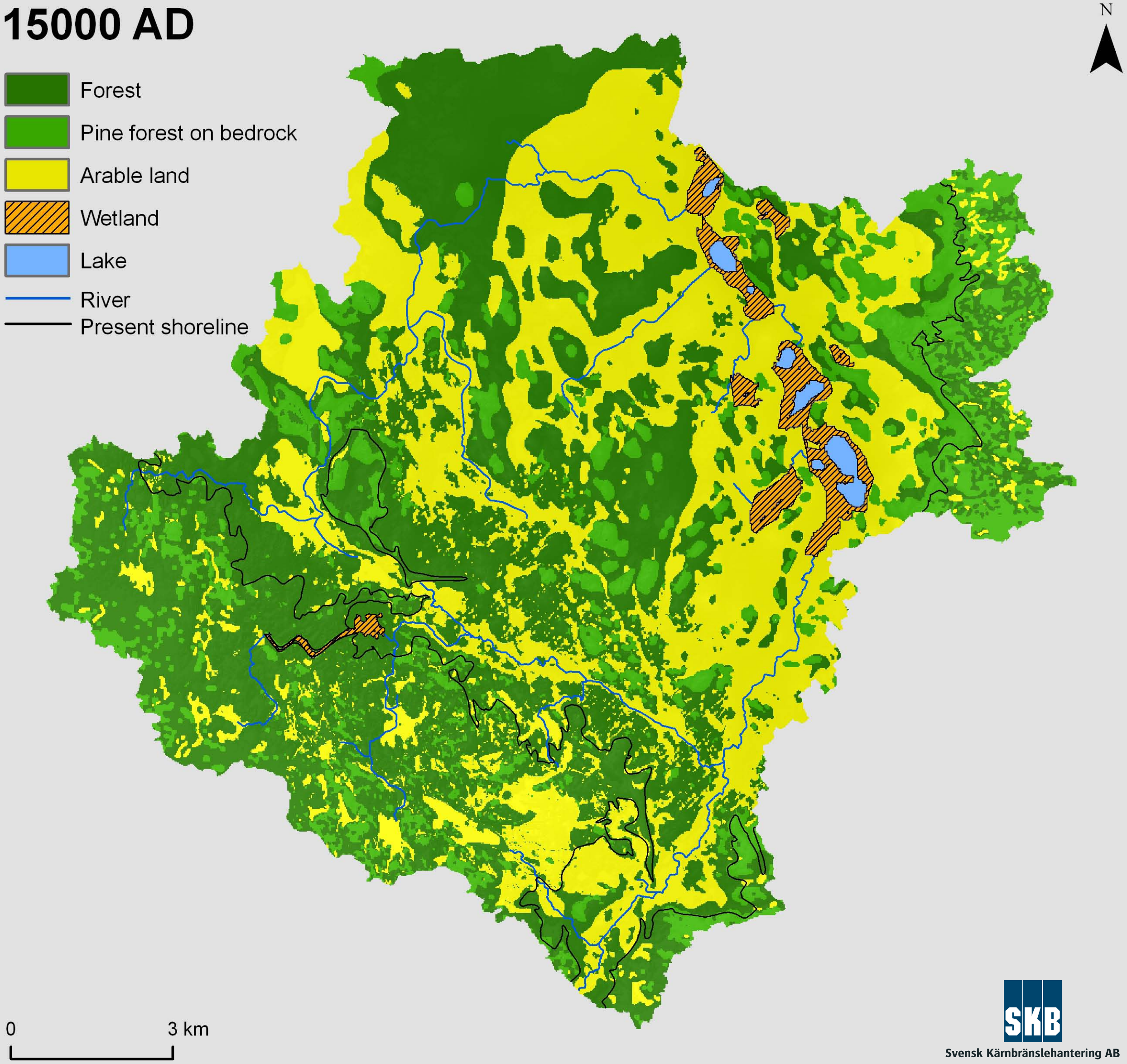
15000 AD
Forest
Pine forest on bedrock
Arable land
Wetland
Lake
River
Present shoreline
N
0
3 km
SKB
Svensk Kärnbränslehantering AB

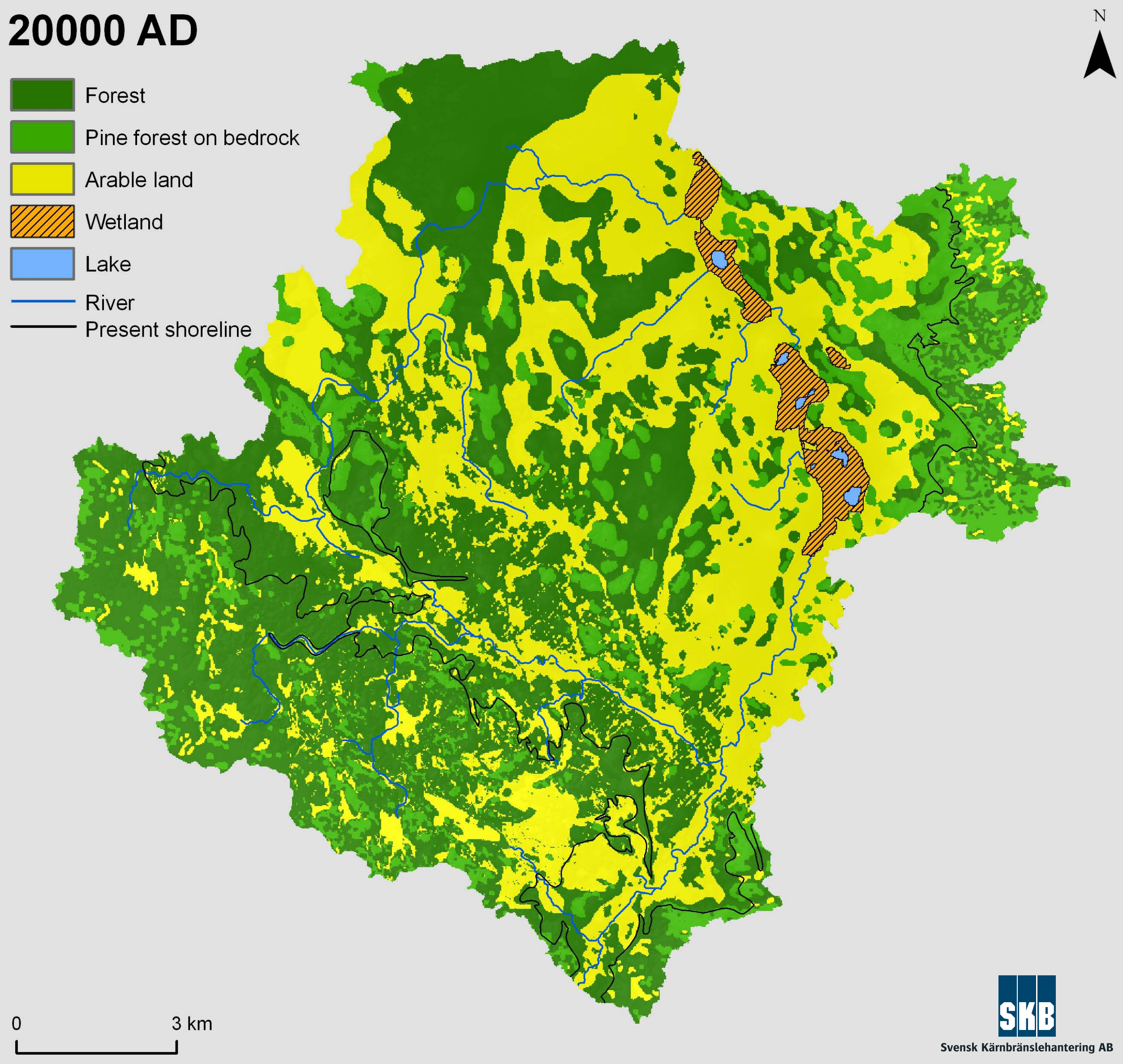

20000 AD
Forest
Pine forest on bedrock
Arable land
Wetland
Lake
River
Present shoreline
N
0
3 km
SKB
Svensk Kärnbränslehantering AB

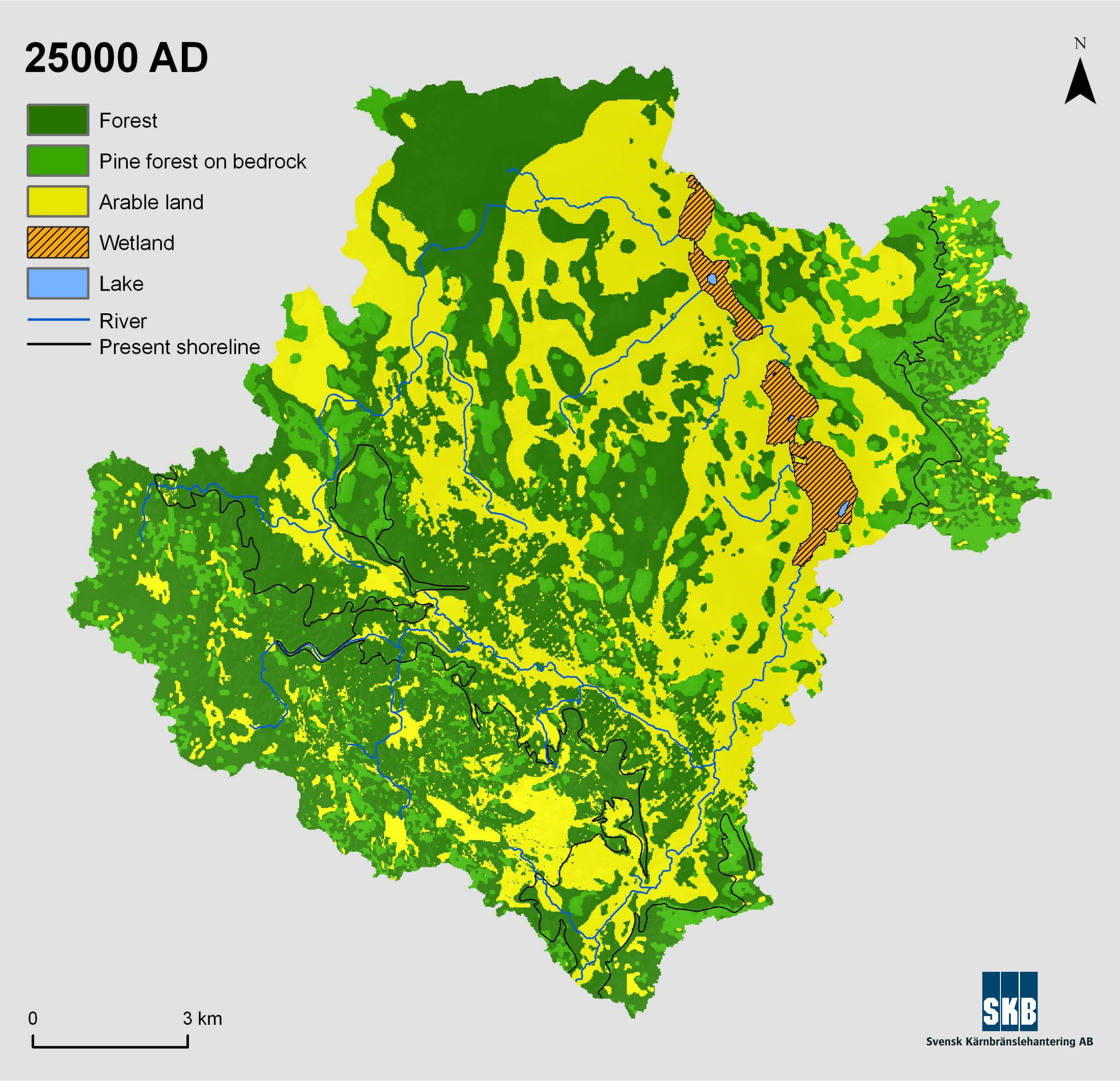
25000 AD
Forest
Pine forest on bedrock
Arable land
Wetland
Lake
River
Present shoreline
N
0
3 km
SKB
Svensk Kärnbränslehantering AB

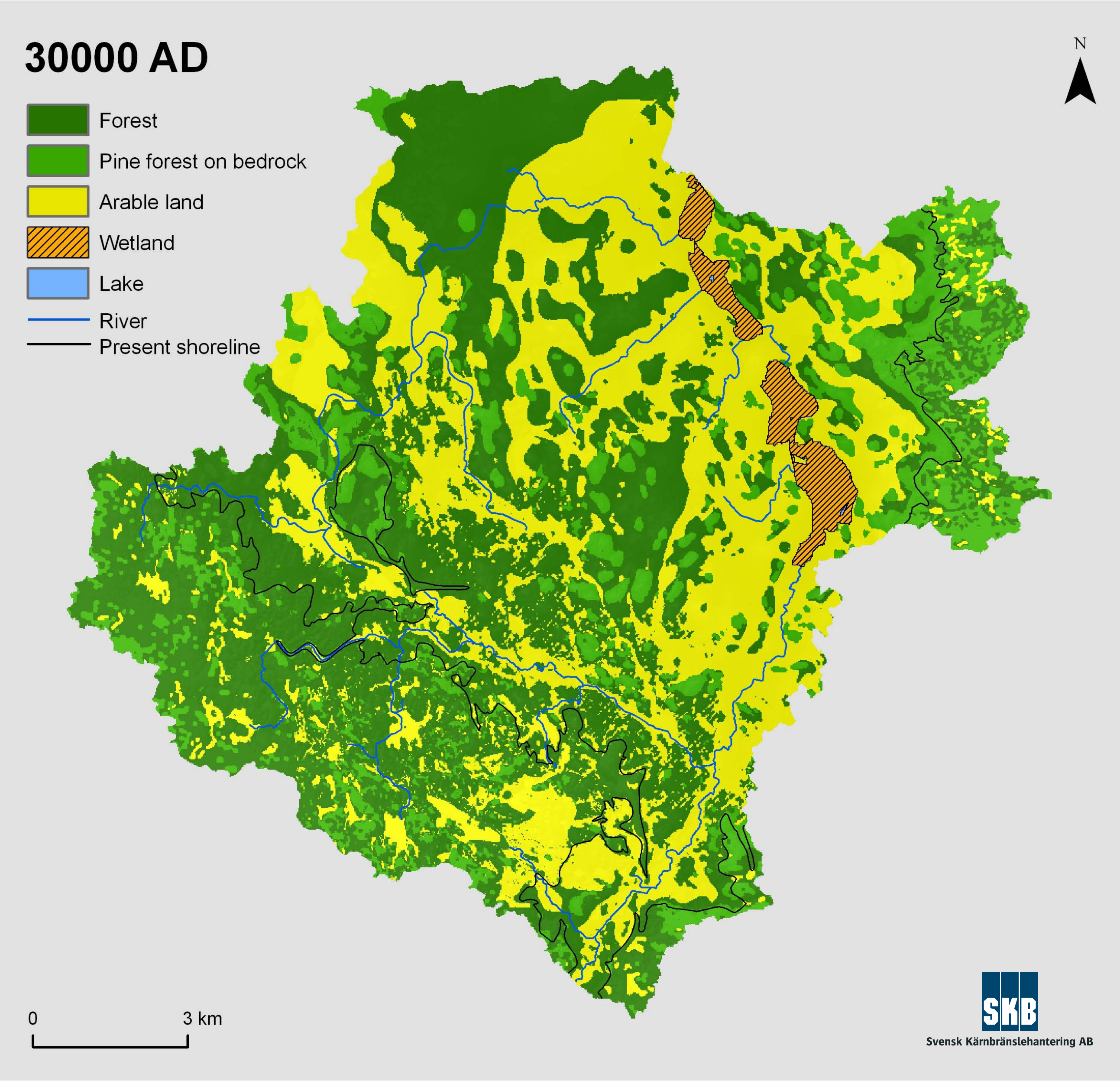
30000 AD
Forest
Pine forest on bedrock
Arable land
Wetland
Lake
River
Present shoreline
N
0
3 km
SKB
Svensk Kärnbränslehantering AB

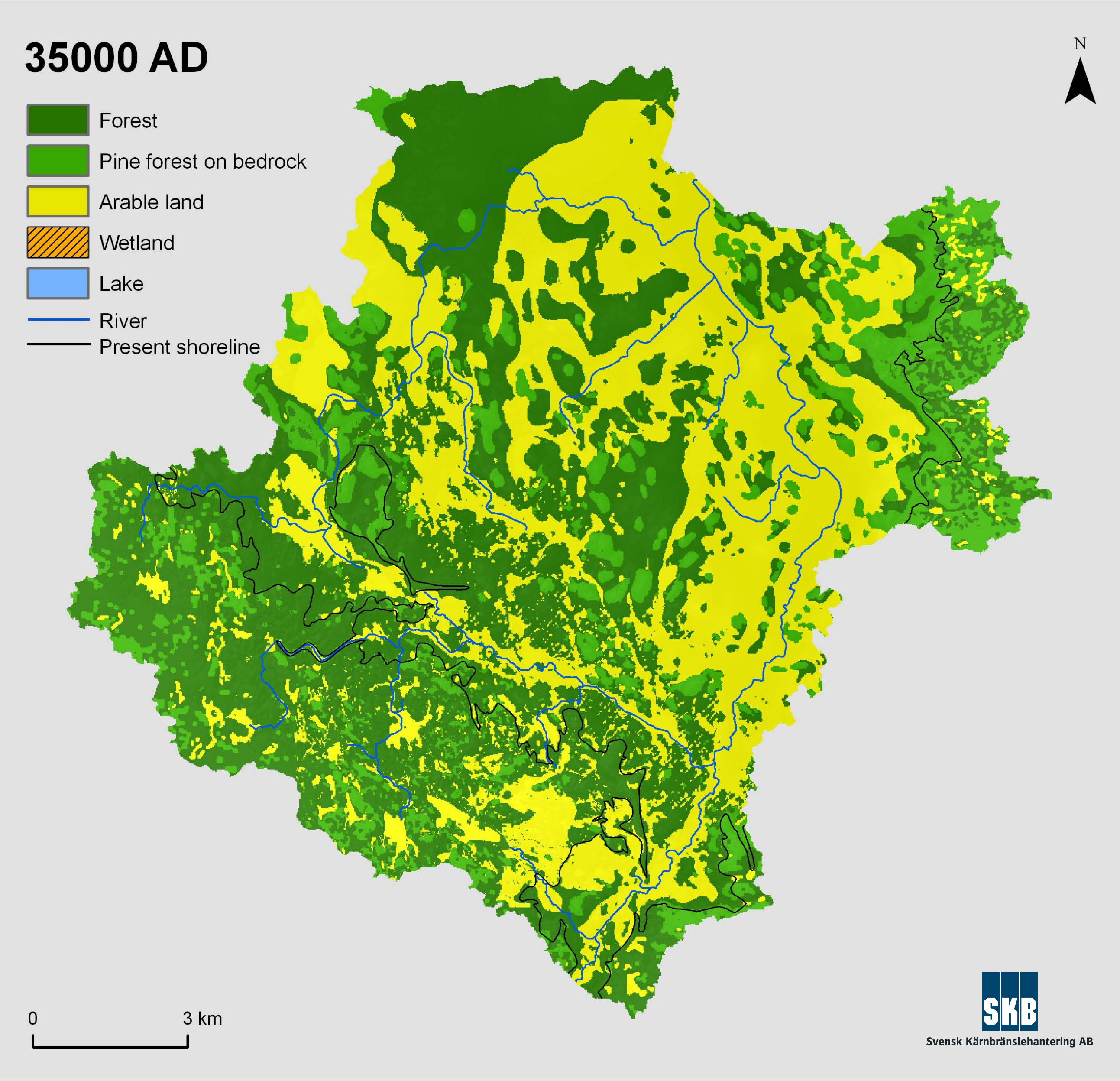
35000 AD
Forest
Pine forest on bedrock
Arable land
Wetland
Lake
River
Present shoreline
N
0
3 km
SKB
Svensk Kärnbränslehantering AB
